# Biomarkers in Peri‐Implant Crevicular Fluid of Healthy Implants and Those With Peri‐Implant Diseases: A Systematic Review and Meta‐Analysis

**DOI:** 10.1111/jop.13612

**Published:** 2025-03-18

**Authors:** Gerardo La Monaca, Nicola Pranno, Romeo Patini, Antonella Polimeni, Massimo Cordaro, Maria Paola Cristalli

**Affiliations:** ^1^ Department of Oral and Maxillo‐Facial Sciences Sapienza, University of Rome Rome Italy; ^2^ Department of Head, Neck and Sense Organs Catholic University of Sacred Heart Rome Italy

**Keywords:** biomarkers, molecular diagnosis, mucositis, peri‐implant crevicular fluid, peri‐implant disease, peri‐implant sulcular fluid, peri‐implantitis

## Abstract

**Introduction:**

Several biomarkers in peri‐implant crevicular fluid have been studied to diagnose peri‐implant diseases with inconclusive results. This systematic review and meta‐analysis aimed to comprehensively compare data on the levels of biological components in peri‐implant crevicular fluid collected from healthy and diseased implants to identify reliable biomarkers for diagnosing and monitoring peri‐implant disease.

**Materials and Methods:**

The search strategy included studies comparing biomarker levels in peri‐implant crevicular fluid between healthy and diseased implants through electronic databases (MEDLINE/PubMed, Embase, Cochrane Library), grey literature, and hand‐searching relevant journals and reference lists of pertinent papers. A two‐stage screening was performed in duplicate and independently. In the first stage, titles and abstracts that fulfilled eligibility criteria were screened. In the second stage, a full‐text analysis was conducted to verify eligibility. All articles meeting the inclusion criteria underwent data extraction and quality assessment. Meta‐analyses were conducted on studies with similar comparisons and outcome measures.

**Results:**

After screening the titles and abstracts, out of 100 potentially relevant papers identified for full‐text evaluation, 49 were excluded, 51 were included in the qualitative analysis, and 18 were included in the quantitative synthesis. Among 96 biomarkers assessed, the most studied were pro‐inflammatory cytokines (IL‐1ß, IL‐6, TNF‐α, and IL‐17), osteoclastogenic‐related factors (RANK, RANKL, and OPG), anti‐inflammatory cytokines (IL‐10), chemokines (IL‐8, MIP‐1α/CCL3, and MIP‐3α/CCL‐20), and enzymes (MMP‐8, Cat‐K, AST, and ALT).

**Conclusions:**

Meta‐analyses comparing data from healthy patients and those with peri‐implantitis or mucositis and between patients with mucositis and those with peri‐implantitis showed a moderate predictive value of IL‐1ß, VEGF, cortisol, and sRANKL/OPG for peri‐implantitis.

## 1 Introduction

For daily clinical practice, the diagnosis of peri‐implant diseases is usually based on the assessment of clinical parameters, including pain, visual signs of inflammation, probing depth, bleeding, suppuration, mobility, and radiographic measures showing progressive changes in crestal bone levels after the initial remodeling process [1]. However, this approach, which relies on clinical and radiographic evaluations, is often ineffective in identifying the disease's onset, activity, and risk rate, potentially hindering early diagnosis and treatment. Peri‐implant probing measurements may be influenced by several factors, such as soft‐tissue biotype, the extent of inflammation, implant geometry and location, prosthetic design, probe type, probing force and direction, and operator accuracy and expertise [2]. Periapical radiographs tend to underestimate peri‐implant marginal bone loss because they detect the mesial and distal aspects around implants, not the 3D morphology of bone defects [3] In addition, some degree of bone loss is required to achieve an accurate measurement, meaning smaller defects are evaluated with less precision than those 3 mm larger [3].

Therefore, to improve this diagnostic protocol, peri‐implant research has proposed an alternative, non‐invasive procedure that assesses host‐derived biomarkers in peri‐implant crevicular fluid (PICF) [4, 5, 6, 7, 8, 9, 10, 11, 12, 13, 14, 15, 16, 17, 18]. Biomarkers are measurable characteristics that indicate normal biological or pathogenic processes or a response to therapeutic intervention. They can monitor health status, detect and stage diseases, indicate disease prognosis, and monitor clinical response to treatment [19]. PICF is a fluid found around dental implants, serving as a serum transudate in healthy implants and an inflammatory exudate in diseased implants. Transudate is a protein‐poor, watery interstitial fluid that results from increased pressure in the veins and capillaries. Exudate, osmotically originating from the gingival plexus vessels, contains micro‐organisms and their products, as well as host‐derived substances such as cytokines, chemokines, bone metabolism markers, enzymes, proteins, and immune system cells, playing a crucial role in the development of mucositis or peri‐implantitis [4, 20].

However, despite numerous studies and reviews focused on analyzing biomarkers in PICF, the efficacy of this approach for predicting, diagnosing, and monitoring peri‐implant inflammatory activity remains undetermined. This systematic review and meta‐analysis aimed to comprehensively compare data on the levels of biological components in PICF collected from healthy and diseased implants, with the goal of identifying reliable biomarkers for diagnosing and monitoring peri‐implant diseases.

## 2 Material and Methods

This systematic review with meta‐analysis was registered in PROSPERO (CRD42024549385) and conducted in accordance with Cochrane Collaboration guidelines [21] and the PRISMA statement [22, 23]. The PRISMA checklist is included in the Supporting Information.

### 2.1 Focused Question

The focused question “Is biomarkers expression in PICF different between healthy implants and those with untreated mucositis and peri‐implantitis, and may it be used for diagnosis?” was formulated according to the PICOS strategy:

POPULATION: Implants with untreated mucositis and/or peri‐implantitis in systemically healthy patients.

INTERVENTION: Biomarker sampling in PICF.

COMPARISON: Healthy implants.

OUTCOMES: Difference in PICF biomarker levels between healthy implants and those with untreated mucositis and/or peri‐implantitis.

STUDY DESIGN: human clinical studies: randomized clinical trials, cross‐sectional studies, case–control studies, controlled clinical trials, cohort studies, case series.

### 2.2 Inclusion Criteria

- Studies on humans reporting levels of any biomarker measured in the PICF of implants with healthy conditions, peri‐implant mucositis, and/or peri‐implantitis, however defined.
- Patients who had not taken antibiotics or anti‐inflammatory drugs within the past 30 days and had not received non‐surgical or surgical treatment for mucositis or peri‐implantitis within the previous 3 months.
- Sample sizes of at least five patients per group.
- All manufacturers, surface types, and implant lengths.
- Articles published in the English language.

### 2.3 Exclusion Criteria

- Case reports, reviews, editorials, letters to the editor, personal opinions, book chapters, theses, short communications, conference abstracts, patents, and pre‐clinical, in vivo, ex vivo, in vitro and animal studies.
- Studies on mini‐implants or implants related to orthodontics and extraoral or zygomatic implants.
- Studies on biomarker evaluation in tissue, serum, saliva, and other biological sources.
- Studies on the assessment of fluid volume, genotypes, or chair‐side tests.
- Unclear criteria for the diagnosis of health and peri‐implant diseases.
- Patients with uncontrolled systemic diseases (cardiovascular, renal or hepatic disease, diabetes, autoimmune deficiency syndrome), a history of chemotherapy or radiotherapy, and pregnancy or lactating.
- Patients who have undergone non‐surgical or surgical treatment for mucositis or peri‐implantitis.

### 2.4 Search Strategy

The search strategy included electronic databases MEDLINE/PubMed, Embase, the Cochrane Library, grey literature, and hand‐searching potentially significant journals in the dental implant field and reference lists of pertinent reviews and selected studies. A comprehensive and systematic electronic search was conducted in the Cochrane Central Register of Controlled Trials (CENTRAL; 2018, Issue 8), MEDLINE via OVID, and EMBASE via OVID from the respective databases' inception to December 2023. The search strategy employed combinations of keywords (Table S1) and was linked with the Cochrane Highly Sensitive Search Strategy (CHSSS) for identifying RCTs in MEDLINE: sensitivity‐maximizing version (2008 revision) as referenced in Chapter 6.4.11.1 and detailed in box 6.4.c of the Cochrane Handbook for Systematic Reviews of Interventions Version 5.1.0.2 2. A hand search limited to articles published between January 2000 and December 2023 was conducted in the peer‐reviewed journals (Table S1). An unpublished literature search was conducted to identify ongoing trials and grey literature in the register of clinical studies of the US National Institutes of Health (www.clinicaltrials.gov) and the multidisciplinary European database (www.opengrey.eu). The references of all retrieved papers and review articles were also checked to select potentially relevant additional studies and to improve the search's sensitivity.

### 2.5 Study Selection

A two‐stage screening was performed in duplicate and independently by two authors (G.L.M. and N.P.). Any disagreement was resolved by discussion, and a third experienced author (M.P.C.) was consulted if necessary. At each of the two‐stage screenings, the inter‐examiner agreement was calculated using Cohen's kappa scores (excellent = *k* > 0.75, fair to good = *k* 0.40–0.75, poor = *k* < 0.40). In the first stage, titles and abstracts fulfilling the eligibility criteria were screened, including abstracts providing unclear, incomplete, or missing information to avoid excluding potentially pertinent publications. In the second stage, a full‐text analysis was conducted to verify eligibility. If more studies reported the same findings, the data were included only once. For redundant publications, the most recent and those with long‐term follow‐ups were considered. All articles meeting the inclusion criteria underwent data extraction and quality assessment. Irrelevant articles were excluded, and the reasons for exclusion were outlined in Table S2.

### 2.6 Data Extraction

An author (G.L.M.) extracted and recorded data from eligible studies on predefined data‐collection tables for qualitative, descriptive, and quantitative analyses. Two other authors (N.P. and R.P.) cross‐checked the accuracy of the registered data. The study characteristics were recorded: first author, year of publication, country, study design, number of patients and/or implants, mean age and gender, functional loading time, case definitions, assessed biomarkers, PICF sample collection, assay methods, biomarker levels, and main results.

### 2.7 Quality Assessment

Two authors (N.P. and R.P.), independently and in duplicate, evaluated the included studies' risk of bias as good, fair, or poor using the NIH Quality Assessment Tool for Observational Cohort and Cross‐Sectional Studies. Due to the purpose of this study, out of 14 questions, number 3 (Was the participation rate of eligible persons at least 50%?) was removed as not applicable.

### 2.8 Assessment of Heterogeneity

Heterogeneity was assessed using Review Manager 5 (RevMan current version: 5.3.5). The significance of discrepancies in the treatment effects between the different studies was estimated with Cochran's test for heterogeneity and the *I* ^2^ statistic. The χ^2^ test was used to evaluate the percentage of total variation due to heterogeneity rather than chance. Heterogeneity was considered significant if χ^2^ < 0.1. The *I* ^2^ statistic was used to quantify inconsistency. An *I* ^2^ value > 50% indicated a moderate to high heterogeneity.

### 2.9 Data Analysis

Meta‐analyses were conducted only for studies with similar comparisons and reporting the same outcome measures. Mean differences were combined for continuous data, using fixed‐effects or random‐effects models, depending on heterogeneity or the number of studies included (fewer than 5). In cases of high heterogeneity, the data were further explored to determine their exclusion from the meta‐analyses. The meta‐analyses were performed according to three main comparisons: implants with peri‐implantitis versus healthy implants (P vs. H), implants with mucositis versus healthy implants (M vs. H), and implants with peri‐implantitis versus implants with mucositis (P vs. M). A forest plot was generated for each meta‐analysis to illustrate the effects of the different studies and the global estimation. In meta‐analyses performed with a fixed‐effects model, funnel plots were created to depict publication bias. All analyses were conducted using Review Manager 5. The significance cut‐off was set at *p*‐value < 0.05. Additionally, each statistically significant meta‐analysis underwent the Trial Sequential Analysis (TSA) software (version 0.9 beta, http://www.ctu.dk/tsa) to correct for alpha and beta errors (alpha error set at 0.05 and beta error set at 20%) and evaluate the power of analysis. TSA software allowed the calculation of the required information size (RIS), the *α*‐spending function, the trial sequential monitoring boundaries for benefits and harms, and futility boundaries. The TSA results were presented in graphs, showing a cumulative *z*‐curve and its relationship with the other curves (trial sequential monitoring boundary, futility boundary and RIS threshold).

## 3 Results

### 3.1 Study Selection

**FIGURE 1:**
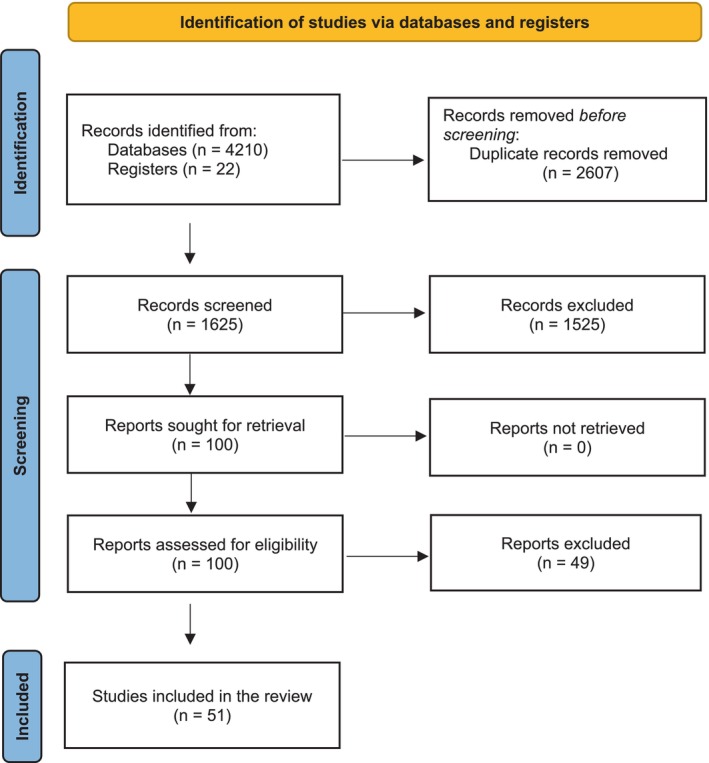
PRISMA 2020 flow diagram of the study selection process.

Figure 1 summarizes the study selection process. Out of 4232 identified articles (4210 through the electronic search and 22 from the hand search), 1625 were evaluated after duplicates were detected and eliminated using EndNote Web—Clarivate Analytics, United States. After screening titles and abstracts, 100 potentially relevant papers were identified for full‐text evaluation. Of the evaluated studies, 49 were excluded because they did not fulfill the inclusion criteria or had unclear information (reasons for exclusion in Table S2). Therefore, 51 studies were included in the qualitative analysis and 18 in the quantitative synthesis. Cohen's *κ* value for the global inter‐reviewer agreement was almost perfect at 0.87 (94.8% agreement).

### 3.2 Biomarkers

The 96 biomarkers assessed included 29 pro‐inflammatory cytokines, 13 anti‐inflammatory cytokines, 10 chemokines, 6 enzymes, and 38 other mediators of inflammation and immunity (Table 1). The number of biomarkers varied across different studies, ranging from 1 in 14 studies, 2 in 12 studies, 3 in 10 studies, 4 in 4 studies, 5 in 6 studies, and between 6 and 27 in 5 studies.

**TABLE 1:** Biomarkers assessed.

Cytokines Pro‐inflammatory	Interleukin (IL)‐1α, ‐1ß, ‐3, ‐5, ‐6, ‐17, ‐17A, ‐17E, ‐20, ‐23; Precursor form of interleukin 1 beta (Pro IL‐1ß); Soluble interleukin 6 receptor (sIL‐6R) ‐ α, ‐β; Tumor necrosis factor alpha (TNF‐α); Soluble Receptor activator of nuclear factor kappa B ligand (sRANKL); Receptor activator of nuclear factor kappa‐beta (RANK); Receptor activator of nuclear factor kappa B ligand (RANKL); Osteoprotegerin (OPG); Tumor necrosis factor ligand superfamily member (TNFSF)‐12/TWEAK, ‐13/APRIL, ‐13B/BAFF, ‐14/LIGHT; Soluble tumor necrosis factor receptor (sTNFR) ‐1, ‐2; Interferon (IFN) ‐α2, ‐β, ‐λ1/IL‐29; Chitinase‐3‐ like‐1; Human B cell‐activating factor/B lymphocyte stimulator (BAFF/BLyS), soluble Suppression of tumorigenicity 2 (sST2)
Cytokines anti‐inflammatory	Interleukin (IL)‐2, ‐4, ‐7, ‐10, ‐11, ‐13, ‐15, ‐19, ‐22, ‐35; Interleukin‐1 receptor antagonist (IL‐1ra); Interferone gamma (IFN‐γ); Tumor necrosis factor‐related apoptosis‐inducing ligand (TRAIL)
Chemochine	Interleukin 8 (IL‐8), Macrophage inflammatory protein (MIP)/Chemokine ligand (CCL)‐1α/CCL3, ‐3α/CCL‐20; Macrophage‐derived chemoattractant (MDC); Monocyte chemoattractant protein (MCP)‐3, ‐1/CCL2; Granulocyte colony‐stimulating factor (G‐CSF); Eotaxin; Chemokine (C‐X‐C motif) ligand 2 (CXCL2); Regulated upon Activation Normal T Cell Expressed and Presumably Secreted (RANTES)
Enzymes	Matrix metallo‐proteinase 8 (MMP‐8); Matrix metallo‐proteinase (MMP‐13); Aspartate aminotransferase (AST); Alkaline phosphatase (ALP); Elastase (EA); Cathepsin K (Cat‐K)
Others	Interleukin (IL) ‐12p40, ‐12p70; Prostaglandin E_2_ (PGE_2_); a2‐Macroglobulin (α2‐M); Transforming growth factor alpha (TGF‐α); Plasminogen activator inhibitor type 2 (PAI‐2); Pyridinoline cross‐linked C‐telopeptide of Type I collagen (ICTP); Osteocalcin (OC); Osteopontin; Osteonectin; Vascular endothelial growth factor (VEGF); Immunoglobulin A1 (IgA1); Calprotectin; Sclerostin; Tissue inhibitor of metalloproteinase 2 (TIMP‐2); Fibroblast growth factor (FGF) ‐2, 23; Platelet‐derived growth factor AB/BB (PDGF‐AB/BB); Granulocyte–macrophage colony stimulating factor (GM‐CSF); Micro RNA (miRNA) ‐21‐3p, ‐150‐5p, ‐26a‐5p; Fms‐like tyrosine kinase‐3 ligand (Flt‐3L); Soluble human CD40 ligand (sCD‐40L); High Mobility Group Box 1 (HMGB1); Substance‐P (SP); Neurokinin‐A (NKA); Calcitonin gene‐related peptide (CGRP); Neuropeptide‐Y (NPY); Procalcitonin (PCT); Extracellular vesicles (EVs); Micro‐vesicles (MVs); Exosomes (Exo); Cortisol (CLs); Neurogenic locus notch homolog protein 1 (NOTCH1); Programmed death‐ligand 1 (PD‐L1/B7‐H1); N‐telopeptide of Type I Collagen (NTx); hypersensitive C‐reactive protein (hs‐CRP); 25(OH)D_3_

### 3.3 Study Characteristics

The main characteristics and findings of the included studies were summarized in Table S3.

The publication years ranged from 1995 to 2023, and the countries involved were Europe, the United States, Brazil, Chile, China, India, Iran, Saudi Arabia, Kuwait, and Turkey. The prevalent study design was cross‐sectional (n.43), followed by case–control (n.5), analytical (n.1) and cohort (n.1).

Most articles reported patient and implant numbers [24, 25, 26, 27, 28, 29, 30, 31, 32, 33, 34, 36, 38, 40, 42, 43, 44, 45, 46, 47, 48, 49, 50, 51, 52, 53, 54, 55, 57, 58, 60, 61, 62, 63, 64, 65, 66, 67, 68, 69, 70, 71, 72, 74]; some indicated only the number of patients [35, 41, 56, 59, 73] or the number of implant sites [37, 39].

All studies, except for four [25, 26, 44, 70], provided the sample's demographic characteristics (age and gender).

Biomarker levels were compared between healthy implants and those with peri‐implantitis in 28 studies [24, 25, 26, 28, 31, 32, 33, 34, 36, 38, 40, 41, 46, 47, 48, 49, 50, 53, 54, 58, 61, 62, 64, 67, 68, 70, 71, 72], healthy implants and those with mucositis in five studies [29, 35, 43, 44, 65], and implants with mucositis and those with peri‐implantitis in 18 studies [27, 30, 37, 39, 42, 45, 51, 52, 55, 56, 57, 59, 60, 63, 66, 69, 73, 74]. All studies, except for seven [39, 42, 43, 59, 67, 69, 70], reported functional loading times ranging from 3 months to 15 years.

There was wide heterogeneity in defining peri‐implant health and disease conditions. In most studies, implant health was defined as the absence of bone loss and signs of inflammation, mucositis as the presence of inflammation without bone loss, and peri‐implantitis as the presence of bone loss along with clinical signs of inflammation (bleeding on probing, suppuration) and a probing depth of 4 mm or more.

Clinical and radiographic parameters were reported in all studies, with a few exceptions [24, 26, 31, 32, 36, 46, 59]. Information on smoking status was not provided in 15 papers [24, 25, 26, 27, 28, 29, 30, 31, 34, 35, 36, 37, 44, 70, 74]. Smokers were excluded in 23 studies [32, 33, 38, 39, 40, 41, 42, 45, 46, 48, 51, 53, 54, 55, 56, 57, 60, 64, 65, 66, 67, 71, 73], and included in 12 [43, 47, 49, 50, 52, 58, 59, 61, 62, 63, 68, 69]. In one article [72], only severe smokers (> 20 cigarettes/day) were excluded.

Most studies failed to provide details regarding a patient's periodontal history [25, 26, 27, 28, 29, 30, 31, 32, 34, 35, 36, 37, 38, 39, 44, 45, 46, 49, 50, 52, 53, 54, 56, 57, 60, 61, 64, 65, 66, 68, 69, 70, 71, 72, 73, 74]. Patients with a history of periodontal disease were included in seven papers [24, 42, 48, 55, 59, 62, 63] and excluded in three papers [33, 51, 67]. Two studies [40, 41] focused on patients with healthy periodontal tissues or those who had received previous periodontal treatment, whereas two studies [43, 47] excluded only patients with uncontrolled periodontal diseases. In one paper [58], patients with chronic, generalized moderate to severe periodontitis and one smoker were included in the peri‐implantitis group.

### 3.4 PICF Sample Collection and Assay

Several methods were used to collect PICF samples and assess biomarkers (Table S3). Nonetheless, all investigations followed the same protocol before proceeding with sampling. The PICF was collected before any probing procedures or the day after the clinical evaluation to avoid blood contamination or other potential alterations. The implants were cleaned of supragingival plaque using sterile curettes or cotton pellets, isolated with cotton rolls, and dried with a gentle air stream for 5–10 s to prevent contamination from saliva. Samples that were visibly contaminated with blood or saliva were discarded.

The most common method for collecting PICF involved inserting sterile periopaper strips into the crevicular sulcus for 30 s. Other techniques included endodontic paper points, microcapillary pipettes, sample syringes with blunt needles, capillary tubes, and point‐of‐care (PoC) dipsticks. Certain studies quantified the absorbed volume of PICF using a Periotron (6000/8000) device. Different methodologies were employed to determine the amount or concentration of biomarkers. The most common methods were ELISA, flow cytometry, Luminex, and spectrophotometry.

### 3.5 Quality and Heterogeneity Assessment

**FIGURE 2:**
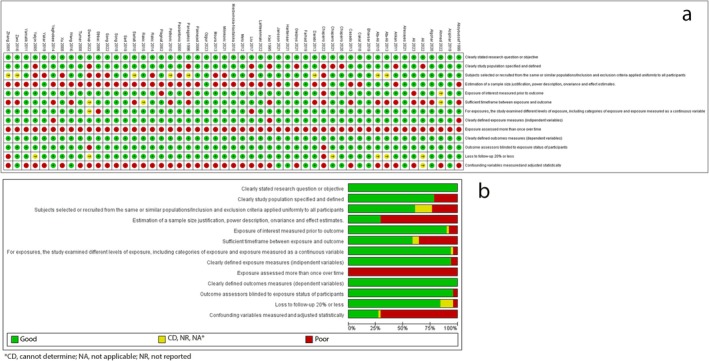
Risk of bias: (a) for each included study; (b) percentages across all included studies.

Figure 2 summarizes the evaluation of bias risks in the included studies. The main strengths were the clarity of the research aim and the measured outcomes. Few studies reported calculating sample sizes and statistically evaluating potential confounding factors, and none assessed the concentration of PICF biomarkers more than once. All meta‐analyses showed high heterogeneity except for sRANKL/OPG and cortisol.

### 3.6 Meta‐Analyses and Trial Sequential Analyses

**FIGURE 3:**
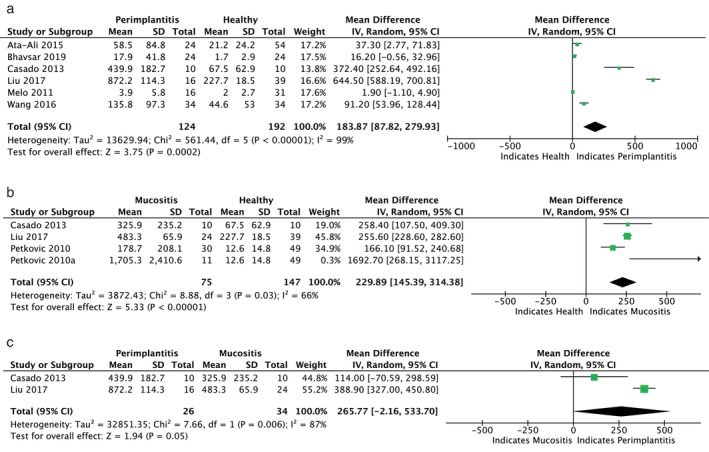
Forest plots presenting standard mean difference (SMD) of IL‐1β comparing: (a) P with H; (b) M and H; (c) P with M.

**FIGURE 4:**
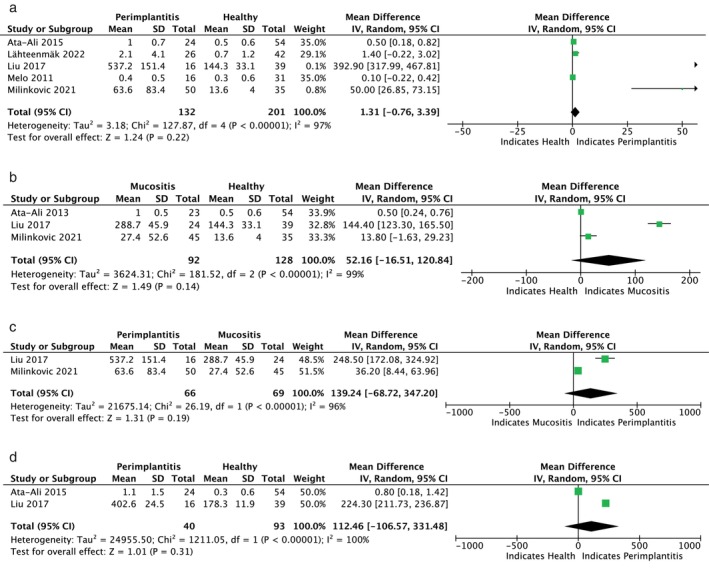
Forest plots presenting standard mean difference (SMD): (a) of IL‐6 comparing P with H; (b) of IL‐6 comparing M and H; (c) of IL‐6 comparing P with M; (d) of TNF‐α comparing P with H.

**FIGURE 5:**
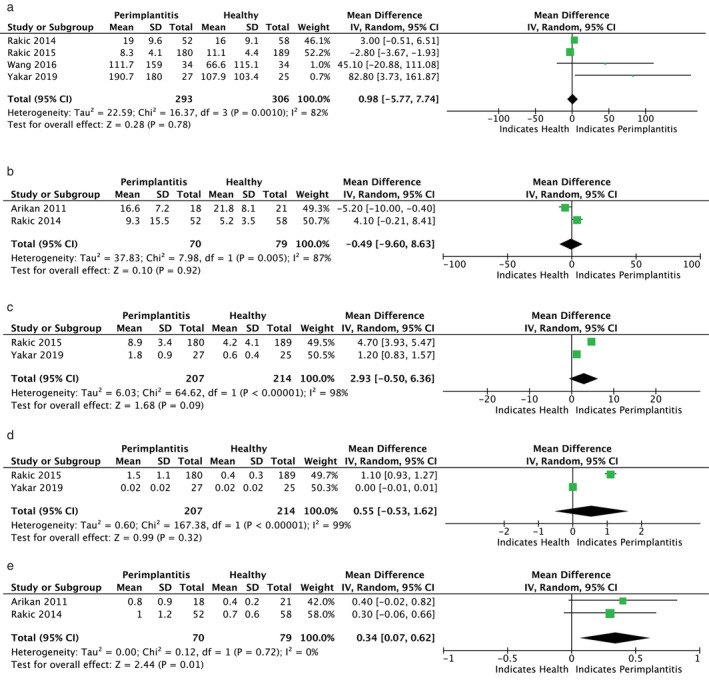
Forest plots presenting standard mean difference (SMD comparing P with H): (a) of OPG; (b) of sRANKL; (c) of RANKL; (d) of RANKL/OPG; (e) of sRANKL/OPG.

**FIGURE 6:**
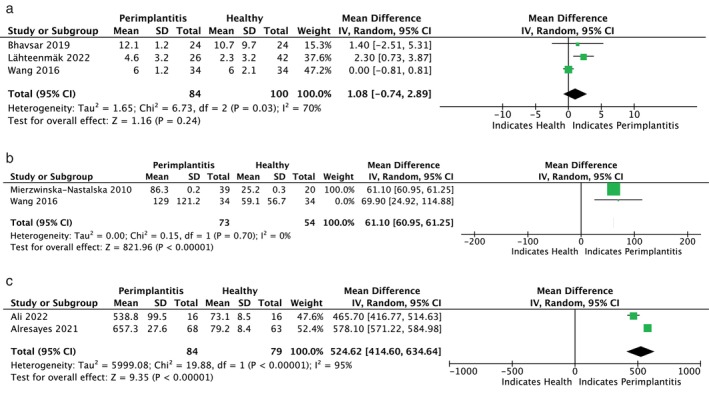
Forest plots presenting standard mean difference (SMD comparing P with H): (a) of MMP‐8; (b) VEGF; (c) of cortisol.

**FIGURE 7:**
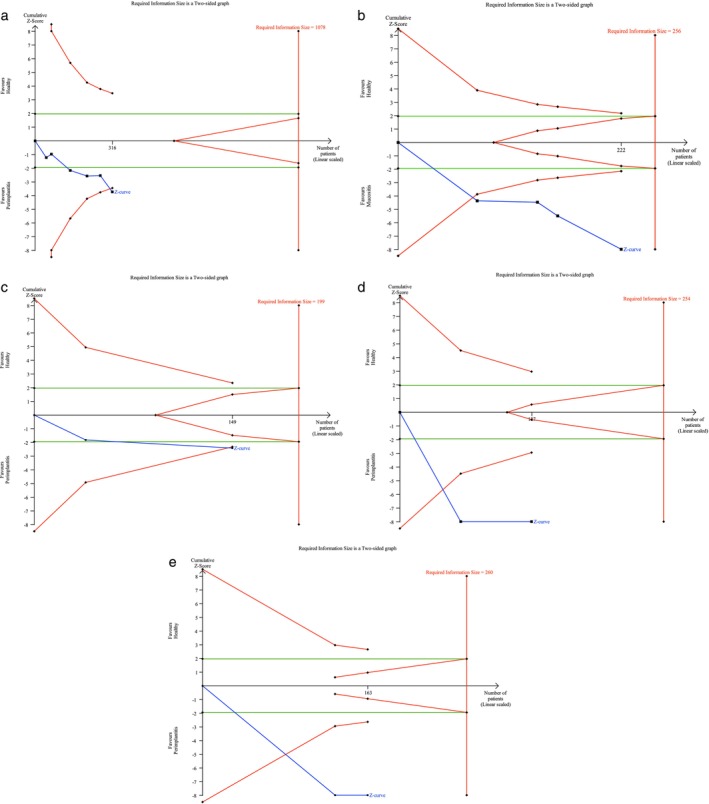
Trial Sequential Analysis (TSA) of the comparisons of PICF concentrations of: (a) IL‐1β (P vs. H); (b) IL‐1β (M vs. H); (c) sRANKL/OPG (P vs. H); (d) VEGF (P vs. H); (e) cortisol (P vs. H).

Figures 3, 4, 5, 6 present meta‐analyses of the available data. Eleven meta‐analyses compared the levels of IL‐1β, IL‐6, TNF‐α, OPG, s‐RANKL, RANKL, sRANKL/OPG, RANKL/OPG, MMP‐8, VEGF, and cortisol in patients with peri‐implantitis and healthy controls. Due to the few available studies, meta‐analyses comparing patients with mucositis to healthy controls and patients with peri‐implantitis to those with mucositis were conducted only for IL‐1β and IL‐6. Nonetheless, the TSA analysis indicated that the evidence from the statistically significant meta‐analyses could not be considered reliable, as the z‐curve crossed the alpha‐spending function and the conventional boundary without reaching the RIS threshold (Figure 7).

According to the GRADE system, the certainty regarding the conclusion and the strength of the evidence from the meta‐analyses on the difference in biomarker concentrations in PICF between healthy and untreated diseased implants was rated as low for VEGF and very low for all other biomarkers. The rating is due to several factors, including study design (case–control, cohort, and cross‐sectional), risk of bias, high heterogeneity, and, in some cases, the wide confidence intervals and the need to convert units of measurement (Table 2).

**TABLE 2:** GRADE summary of meta‐analysis outcomes on differences in biomarker concentration in PICF between healthy implants and those with mucositis and periimplantitis.

Quality Assessment, Outcome: concentration of biomarkers in PICF of implants affected by mucositis, peri‐implantitis and healthy ones						
Question: Is the expression of the biomarkers in PCIF different between implants in healthy and untreated diseased conditions?						
Number of studies according to meta‐analysis (n.)	Study design	Risk of bias	Inconsistency	Indirectness	Imprecision	Publication bias
n. 6 Figure 3a—IL‐1β P vs. H	C‐c/C‐s/Cohort	S	S^1^	No S	No S	U
n. 4 Figure 3b—IL‐1β M vs. H	C‐c/C‐s/Cohort	S	S^1^	No S	S^3^	U
n. 2 Figure 3c—IL‐1β P vs. M	C‐c/C‐s/Cohort	S	S^1^	No S	S^3^	U
n. 5 Figure 4a—IL‐6 P vs. H	C‐c/C‐s/Cohort	S	S^1^	No S	No S	U
n. 3 Figure 4b—IL‐6 M vs. H	C‐c/C‐s/Cohort	S	S^1^	No S	No S	U
n. 2 Figure 4c—IL‐6 P vs. M	C‐c/C‐s/Cohort	S	S^1^	No S	No S	U
n. 2 Figure 4d—TNF‐α P vs. H	C‐c/C‐s/Cohort	S	S^1^	No S	No S	U
n. 4 Figure 5a‐ OPG P vs. H	C‐c/C‐s/Cohort	S	S^1^	No S	S^3^	U
n. 2 Figure 5b‐ sRANKL P vs. H	C‐c/C‐s/Cohort	S	S^1^	No S	No S	U
n. 2 Figure 5c‐ RANKL P vs. H	C‐c/C‐s/Cohort	No S	S^1^	No S	No S	U
n. 2 Figure 5d—RANKL/OPG P vs. H	C‐c/C‐s/Cohort	S	S^1^	No S	No S	U
n. 2 Figure 5e—sRANKL P vs. H	C‐c/C‐s/Cohort	S	No S	No S	S^3^	U
n. 3 Figure 6a—MMP‐8 P vs. H	C‐c/C‐s/Cohort	No S	S^1^	S^2^	No S	U
n. 2 Figure 6b—VEGF P vs. H	C‐c/C‐s/Cohort	No S	No S	No S	No S	U
n. 2 Figure 6c—Cortisol P vs. H	C‐c/C‐s/Cohort	S	S^1^	No S	No S	U

Abbreviations: 1 = due to high heterogeneity; 2 = due to converting units of measure; 3 = due to wide confidence intervals; C = case–control; C‐s = Cross‐sectional; S = serious; U = undetected.

## 4 Discussion

Several reviews [4, 5, 6, 7, 8, 9, 10, 11, 12, 13, 14, 15, 16, 17, 18] have focused on assessing specific PICF biomarkers involved in the immune‐inflammatory processes of peri‐implant diseases. However, to the authors' knowledge, no comprehensive systematic analysis of all biomarkers studied in the literature has been conducted. This systematic review and meta‐analysis summarized direct comparisons of biomarker levels in the PICF of healthy implants and those with untreated mucositis and/or peri‐implantitis. The goal was to identify reliable biomarkers for diagnosing and monitoring peri‐implant diseases, highlighting the potential of these markers for complementing clinical and radiographic diagnosis.

### 4.1 Pro‐Inflammatory Cytokines

Pro‐inflammatory cytokines are the most extensively studied biomarkers due to their crucial role in activating cellular and vascular inflammatory responses, focusing on IL‐1β, IL‐6, TNF‐α, and IL‐17 [75].

IL‐1ß, produced by host monocytes and macrophages in response to bacterial products, induces the release of various inflammatory mediators, resulting in a cascade of events that leads to soft and hard tissue destruction. When patients with mucositis were compared to healthy controls, Petković et al. [35] found significantly higher levels of IL‐1β in implants with mucositis and in advanced cases than in early mucositis, unlike Ata‐Ali et al. [43], who failed to show a statistically significant difference. IL‐1β levels were also generally higher in peri‐implantitis than in healthy implants and in the early stages than in advanced stages [24, 25, 46, 47, 49, 50, 58, 61, 71, 72]. Increasing IL‐1β levels in early peri‐implantitis, probably due to the acute nature of this condition versus the more chronic nature of the advanced disease, suggested that this cytokine may be a reliable biomarker for prompt diagnosis [25, 26]. Two studies [42, 51] corroborated these findings, detecting lower levels of IL‐β in healthy sites than in those affected by mucositis and peri‐implantitis and in mucositis versus peri‐implantitis implants. Nevertheless, the lack of a significant difference between the two diseased groups prevented the differentiation of peri‐implant mucositis from peri‐implantitis. Meta‐analyses confirmed the link of IL‐1β increased concentrations with peri‐implant diseases, reporting statistically significant mean differences between 124 patients with peri‐implantitis and 75 with mucositis compared with 192 and 147 healthy subjects, respectively [35, 42, 51].

IL‐6 promotes pro‐inflammatory responses to bacterial infections, enhancing T‐lymphocyte proliferation and B‐lymphocyte differentiation, activating the complement cascade, and inducing bone resorption [76]. Significantly greater expression of IL‐6 was found in implants with mucositis [30, 43, 51, 66, 73] and peri‐implantitis [46, 47, 54, 68, 72] compared to healthy ones and in peri‐implantitis versus mucositis sites [30, 50, 66, 73]. However, two studies [40, 61] detected similar concentrations in the peri‐implantitis and control groups. The meta‐analysis did not reveal significant differences in IL‐6 levels between healthy individuals and those with peri‐implant diseases.

TNF‐α is mainly synthesized by mononuclear macrophages and is strongly linked to disease progression. Its role in increasing marrow osteoclast precursors leads to bone resorption. Conflicting results on TNF‐α were reported. Some studies showed significantly higher levels of TNF‐α in the mucositis [35, 51, 65] and peri‐implantitis groups [41, 47, 50, 51, 54, 71] than in healthy groups, while others observed no comparable levels in peri‐implantitis and healthy implants [61, 72]. The meta‐analysis, which compared data from two studies [47, 51] involving 40 peri‐implantitis patients and 93 healthy controls, found no statistical significance between the groups.

IL‐17 links T‐cell activation to neutrophil mobilization and activation, playing a key role in innate immunity, inflammation, and osteoclastogenesis [77]. The average amount of IL‐17 in the PICF of implants with mucositis [56, 65] and peri‐implantitis [41, 50, 56, 66] was significantly higher than in the control groups. Contradictory results emerged when comparing mucositis and peri‐implantitis: one study [56] reported lower IL‐17 levels in peri‐implantitis than in mucositis, while another [66] showed higher levels.

### 4.2 Osteoclastogenesis‐Related Factors

Factors related to osteoclastogenesis are crucial in the processes of bone resorption, influencing the development and progression of peri‐implantitis.

RANK, RANKL, and sRANKL are proteins that stimulate osteoclast precursors to initiate bone resorption. These factors were statistically increased in implants affected by peri‐implantitis compared to healthy implants [45, 48, 53, 59] and in sites with mucositis versus those with peri‐implantitis [59, 69] Nevertheless, two studies yielded inconsistent data, showing either a higher sRANKL concentration in healthy individuals than in peri‐implantitis patients [38] or no statistically significant difference [37].

OPG is a decoy receptor for RANKL and counterbalances sRANKL by inhibiting osteoclast differentiation and activity. Results varied among different studies, reporting higher concentrations of OPG in peri‐implantitis [49, 53] and mucositis [69] than in healthy sites [38, 48], or comparable amounts across all three groups [61]. These contradictory findings suggest that OPG may fluctuate based on the inflammatory environment and patient‐specific conditions.

Most studies [38, 45, 59] found no significant differences in the sRANKL/OPG relative ratio between healthy and diseased implants, except for one [48] that detected higher values in the peri‐implantitis group.

None of the meta‐analyses conducted on RANKL, s‐RANKL, OPG, and RANKL/OPG demonstrated statistically significant results. Only a meta‐analysis involving 70 peri‐implantitis patients and 79 healthy individuals [38, 45] supported the predictive value of increased sRANKL/OPG levels for peri‐implantitis.

### 4.3 Anti‐Inflammatory Cytokines

Anti‐inflammatory cytokines are immunoregulatory molecules that inhibit inflammation and the production of pro‐inflammatory cytokines and regulate immune cell activation and functions [78]. The most investigated was IL‐10, which is produced by T and B lymphocytes, activated monocytes, and macrophages and has immunosuppressive properties that inhibit the synthesis of pro‐inflammatory cytokines [78]. The IL‐10 concentration, typically elevated in patients with peri‐implant diseases compared to healthy subjects and in patients with mucositis rather than peri‐implantitis, suggested a relationship with the peri‐implant tissue inflammatory response [47, 50, 56]. However, these findings were inconsistent with two studies reporting higher IL‐10 levels in healthy implants [42] and no significant difference between healthy and diseased sites [72].

### 4.4 Inflammatory Chemokines

Inflammatory chemokines, such as IL‐8, MIP‐1α/CCL3, and MIP‐3α/CCL‐20, act mainly by attracting monocytes, neutrophils, and other effector cells from the blood to sites of infection or tissue damage.

IL‐8 is produced by multiple cell types, including macrophages, epithelial cells, and mesenchymal stem cells. Its role in activating osteoclasts is linked to progressive bone loss over time. Compared to healthy implants, significantly elevated IL‐8 concentrations were observed in peri‐implantitis [62, 72] and mucositis [51] implants. These levels were higher in the early stages than in the advanced stages [35], without any difference between the diseased sites. These findings, showing the biomarker's capacity to improve diagnostic specificity, conflicted with the results of one study, which reported no significant differences between healthy and diseased implants, although IL‐8 increased in the peri‐implantitis group [47].

MIP‐1α/CCL3 is a macrophage inflammatory protein that induces an inflammatory response characterized by neutrophil infiltration and promotes osteoclastogenesis through the direct activation of osteoclasts. The potential diagnostic role of this chemokine in distinguishing peri‐implant health from disease remains controversial. Only one study showed an increase in diseased sites compared to healthy ones [35], while others [58, 62, 72] found no difference.

MIP‐3 α/CCL‐20 is a strong chemotactic agent for B and T lymphocytes and a weak neutrophil attractant that coordinates and regulates humoral immune responses [79]. The average concentration was lowest in healthy sites, increased during the initial inflammatory phase of mucositis, and decreased to about half in peri‐implantitis [69], with no significant differences between healthy and diseased implants [59, 69, 72].

### 4.5 Enzymes

Among the enzymes involved in periodontal and peri‐implant diseases, MMP‐8, Cat‐K, AST, and ALP were the most extensively studied, whereas EA28 and MMP‐1354 were evaluated only once and in very small samples.

MMP‐8 (also known as collagenase‐2 or neutrophil collagenase) plays a crucial role in peri‐implant tissue destruction, promoting the initiation of inflammatory collagen destruction, particularly types I and III. According to the Authors, who showed increasing collagenase activity in implants with peri‐implantitis [33, 54, 61, 68] and mucositis [65] compared to healthy implants, the assessment of MMP‐8 in PICF served as a reliable diagnostic test to differentiate between peri‐implant health and disease. On the contrary, in studies [49, 58], which reported no significant differences between the healthy and peri‐implantitis groups, this proteolytic enzyme was deemed an unreliable indicator of peri‐implant disease. The meta‐analysis of data from three studies [49, 58, 68], including 84 patients with peri‐implantitis and 100 healthy patients, showed no statistical significance.

Cat‐K is a protease highly expressed in osteoclasts. It is released in its active form into the pericellular space to degrade matrix proteins, such as collagen type I, osteopontin, and osteonectin, under acid pH conditions [32, 45]. The total amount of Cat‐K and the PICF volume were higher in peri‐implantitis than in healthy peri‐implant tissues and were positively correlated with clinical parameters [32]. Nevertheless, when normalized to PICF volume, Cat‐K levels demonstrated no significant difference between the two groups and a slightly negative correlation with clinical parameters. Therefore, combining PICF volume sampling with immunoassay was considered unsuitable for detecting peri‐implantitis, as this method provided no extra information about the disease's status beyond volume measurements alone. Conflicting opinions were reported about the significance of the Cat‐K increase. In one study, significantly higher Cat‐K activity in peri‐implantitis sites than in healthy sites and those with mucositis was explained by inflammation, which, although affecting the enzymatic profile, induced a marked increase only in the presence of alveolar bone loss [39]. In another investigation, significantly higher concentrations in implants with mucositis than in healthy implants, but not between those with peri‐implantitis and mucositis, suggested that Cat‐K may indicate early osteoclastogenesis and the onset of peri‐implantitis [45].

AST is present in the cell cytoplasm and is released by dead or dying cells, while ALP is found on the surface of osteoblasts and reflects their biosynthetic activity. AST activity was significantly greater in peri‐implantitis than in the healthy and mucositis groups, reflecting increased tissue breakdown in the diseased areas [27]. The hypothesis that ALP may be a potential biomarker to predict the onset of peri‐implant disease was supported by findings detecting total amounts six times greater in implants with peri‐implantitis than in healthy ones, along with its correlation to clinical parameters [28]. When data were examined on an individual patient basis, increased BoP scores were significantly associated with AST but not with ALP [31]. This lack of a relationship between AST/ALP activity and clinical parameters was due to the loss of site information resulting from the average of each variable used to evaluate individual patients. Therefore, patients with low AST/ALP levels should not be assumed to have all implants healthy as a diseased site may be present.

### 4.6 Other Biomarkers

Among the other PICF biomarkers analyzed, VEGF, PGE [2], and cortisol were of some interest. VEGF is a pro‐inflammatory mediator that promotes angiogenesis and microvascular permeability in response to cytokines and growth factors. The significant increase in VEGF concentration found around peri‐implantitis sites, in contrast with those clinically healthy [36, 49, 72], would suggest its ability to predict disease status. A meta‐analysis involving 73 patients with peri‐implantitis and 54 in the control group endorsed this predictive value [36, 49]. Conversely, detecting similar levels of VEGF in a multi‐biomarker approach indicated that this biomarker alone might be inadequate to discriminate between peri‐implant health and disease [50]. Nevertheless, its association with IL‐17 and IL‐1ra represented a 3‐biomarker best‐fit model, even for samples collected from clinically healthy sites around diseased implants [50].

PGE_2_, synthesized by macrophages and gingival fibroblasts, exerts pro‐inflammatory effects by inducing vasodilation, increased vascular permeability at inflammation sites, and osteoclastic activity. Conflicting opinions were reported on PGE_2_ as a potential biomarker for assessing peri‐implant status. Two studies showed higher levels in patients with peri‐implant diseases compared to healthy individuals and in implants with peri‐implantitis versus those with mucositis [29, 67]. Instead, in one study, no significant differences were found between healthy sites and those with early peri‐implantitis, and there was no correlation with levels of IL‐1ß in the same samples [26]. Based on these results, PGE [2] did not seem to be stimulated in diseased sites nor contribute to the inflammatory process.

Cortisol is a stress‐related hormone with anti‐inflammatory properties, which is released into the bloodstream in free and protein‐bound forms. Although elevated levels were detected in patients with peri‐implantitis, the predictive value of cortisol remains unclear, as other conditions, including anxiety/depression, tobacco smoking, and chronic hyperglycemia, may affect its production [64, 67].

The above‐reported data failed to provide robust evidence regarding which biomarkers are effective for diagnosing and monitoring peri‐implant diseases due to the heterogeneity and shortcomings of the included studies. The small sample size, lack of power calculation, heterogeneous study population (influenced by different case definitions used to select and group the participants), and differences in reporting confounding factors, such as smoking habits and a history of periodontitis, may have impacted the significance of the results. Other biases involved differences in the methods used to collect and analyze PICF samples and in the presentation of data, such as amounts, total concentration, and mean or median values, which hindered finding comparisons. Additionally, variability in the timing of sample collection relative to implant insertion or functionalization may have played a role in biomarker expression, as implants that have been in place for extended periods are more susceptible to peri‐implant disease than those that have been inserted recently. Lastly, a single moment of PICF collection might not detect the levels of immune‐inflammatory biomarkers, which are not always active due to the cyclic progression of peri‐implant diseases. Indeed, multiple time points are needed to investigate the correlation between peri‐implant bone loss and biomarkers, as bone resorption increases over time following a non‐linear process.

## 5 Strengths and Limitations

This systematic review has strengths and limitations. Strengths were the comprehensive literature search and the dual selection process (computerized and manual), which ensured the inclusion of nearly all relevant papers on PICF biomarker assessment. Also, the meta‐analyses, which integrated diverse and conflicting findings from the individual studies, allowed for a more precise evaluation of the outcomes. Limitations involved the decision to include only studies published in English, which may have led to the exclusion of relevant information, the limited number of studies available for meta‐analysis, and the absence of randomized controlled trials that could have affected the results.

## 6 Conclusions

This review and meta‐analysis summarized the currently available knowledge on PICF biomarkers to aid in identifying repeatable, noninvasive diagnostic tools to diagnose and monitor mucositis and peri‐implantitis. The reported data revealed that, among all the PICF biomarkers analyzed, IL‐1ß, VEGF, cortisol, and sRANKL/OPG seemed to have predictive value for peri‐implantitis, even if the strength of evidence was only moderate. Therefore, additional research is needed to establish the reliability, specificity, and sensitivity of PICF biomarker assessment before this approach can be effectively implemented in clinical practice.

## Author Contributions

Gerardo La Monaca contributed to data acquisition, formal analysis and interpretation, and drafted and critically revised the manuscript; Nicola Pranno contributed to data acquisition, statistics, and drafted the manuscript; Romeo Patini contributed to data acquisition, formal analysis and interpretation, statistics, and drafted and critically revised the manuscript; Antonella Polimeni contributed to the conception and design of the study, data analysis and interpretation, and critically revised the manuscript; Massimo Cordaro contributed to the conception and design of the study, data analysis and interpretation, and critically revised the manuscript; Maria Paola Cristalli contributed to data acquisition, formal analysis and interpretation, and drafted and critically revised the manuscript. All authors gave final approval and agreed to be accountable for all aspects of the work. Gerardo La Monaca, Nicola Pranno, and Romeo Patini contributed equally to this work.

## Conflicts of Interest

The authors declare no conflicts of interest.

### Peer Review

The peer review history for this article is available at https://www.webofscience.com/api/gateway/wos/peer‐review/10.1111/jop.13612.

## Supporting information




**Table S1.** MeSH terms and free text words used in electronic searching and peer‐reviewed journals screened in hand search.

**Table S2.** Summary of excluded studies and reasons for exclusion.

**Table S3.** Main characteristics of the selected studies.

## Data Availability

The data that support the findings of this study are available on request from the corresponding author. The data are not publicly available due to privacy or ethical restrictions.
