# Supplementary material for: Biomarkers in Peri‐Implant Crevicular Fluid of Healthy Implants and Those With Peri‐Implant Diseases: A Systematic Review and Meta‐Analysis
Source: J Oral Pathol Med. 2025 Mar 18;54(5):267–82. doi: 10.1111/jop.13612 (PMC12077954; doi:10.1111/jop.13612)
Supplement: Supplementary file 1 — Table S1. MeSH terms and free text words used in electronic searching and peer‐reviewed journals screened in hand search. [file JOP-54-267-s002.docx]

**Supplementary Table 1**: MeSH terms and free text words used in electronic searching and peer-reviewed journals screened in hand search

| MeSH terms and free text words | [(“dental implant” OR “Surgical Dental Prosthesis” OR “Implants” [MeSH Terms]) OR (“dental implants” OR “osseointegrated implants” [Text Word]) AND (“healthy implants” [Text Word]) OR (“mucositis” [MeSH Terms]) OR (“peri-implant mucositis” OR implant mucositis [Text Word]) OR (“peri-implantitis” [MeSH Terms]) OR (“peri-implantitis” [Text Word]) AND (“inflammation” [MeSH Terms] OR inflammation [Text Word]) AND (“biomarkers” OR “markers” OR “biological marker” OR “immune markers” OR “ serum marker” OR “clinical markers” OR “biochemical markers” [MeSH Terms]) OR (“cytokines” [MeSH Terms]) OR (“chemokines” [MeSH Terms]) OR ("Interleukins"[Mesh]) OR ("Enzymes"[Mesh]) OR (acute phase protein [Text Word]) |
| --- | --- |
| Peer-reviewed journals | Implant dentistry, Clinical Oral Implants Research, Clinical Implant Dentistry and Related Research, European Journal of Oral Implantology, International Journal of Oral & Maxillofacial Implants, International Journal of Oral and Maxillofacial Surgery, Journal of Periodontology, Journal of Clinical Periodontology, International Journal of Periodontics and Restorative Dentistry, Periodontology 2000, Journal of Dental Research, International Journal of Oral Science, Journal of Dentistry, Journal of Periodontal Research, Journal of Evidence-Based Dental Practice And Clinical Oral Investigations, British Journal of Oral and Maxillofacial Surgery, Journal of Periodontology and Implant Dentistry; Journal of Periodontal and Implant Science, Journal of Periodontology and Implant Research, Journal of Oral Implantology, Oral and Implantology |
