# Supplementary material for: Biomarkers in Peri‐Implant Crevicular Fluid of Healthy Implants and Those With Peri‐Implant Diseases: A Systematic Review and Meta‐Analysis
Source: J Oral Pathol Med. 2025 Mar 18;54(5):267–82. doi: 10.1111/jop.13612 (PMC12077954; doi:10.1111/jop.13612)
Supplement: Supplementary file 2 — Table S2. Summary of excluded studies and reasons for exclusion. [file JOP-54-267-s001.docx]

**Supplementary Table 2**: Summary of excluded studies and reasons for exclusion.

| Authors (year)  Country | Biomarkers | Reasons for exclusion |
| --- | --- | --- |
| 1. Adonogianaki et al.  (1995)  Sweden | α2-macroglobulin (α2-M), α1-antitrypsin (α1-AT9), Transferrin (TF), Lactoferrin (LF), Albumin (Alb),  IG against P. ginigivalis | Unclear presentation of the clinical parameters used for diagnosis of healthy and peri-implant diseased groups |
| 2. Salcetti e al.  (1997)  USA | Prostaglandin E2 (PGE2), Interleukin-1ß (IL-1ß) Interleukin 6 (IL-6), Transforming growth factor ß (TGF-ß),  Platelet-derived growth factor (PDGF). | Unclear criteria for the diagnosis of peri-implant disease groups. In the text, it was used the term “failing implants.” |
| 3. Fiorellini et al.  (2000)  USA | Aspartate aminotransferase (AST) | Focus on correlation of AST levels in PICF with GI, Bop, PD around implants |
| 4. Nomura et al.  (2000)  Japan | Tissue inhibitors of metalloproteinases (TIMP-1), Matrix metalloproteinase 1 (MMP-1), Matrix metalloproteinase 8 (MMP-8) | Evaluation in PICF:1) during wound healing after implantation; 2) from implants affected with peri-implantitis without comparison to healthy implants |
| 5. Murata et al.  (2002)  Japan | Osteocalcin, Deoxypyridinoline, Interleukin-1β | Lack of valid data (mean, SD) |
| 6. Ataoglu et al.  (2002)  Turkey | Interleukin-1β (IL-1β), Tumor necrosis factor-alpha (TNF- α), neutrophil elastase (NE) | Unclear presentation of the clinical parameters used for diagnosis of healthy and peri-implant diseased groups |
| 7. Hultin et al.  (2002)  Sweden | Interleukin-1β (IL-1β), lactoferrin (LF), elastase | Lack of valid data (mean, SD) |
| 8. Kivelä-Rajamäki et al.  (2003)  Finland | Laminin-5 2-chain, Matrix metalloproteinase 8 (MMP-8) | Unclear diagnosis of healthy and peri-implant diseased groups, lack of valid data |
| 9. Kivelä-Rajamäki et al.  (2003)  Finland | Matrix metalloproteinase 7 (MMP-7), Matrix metalloproteinase 8 (MMP-8) | Unclear diagnosis of healthy and peri-implant diseased groups, Lack of valid data |
| 10. Liskmann et al.  (2004)  Estonia | Myeloperoxidase (MPO) | Lack of differential diagnosis of mucositis and peri-implantitis. The generic definition "implants with inflammatory lesions" was used |
| 11. Monov et al.  (2006)  Austria | Soluble receptor activator of nuclear factor kappa-B ligand (sRANKL), Osteoprotegerin (OPG) | No distribution into H, M, and PI groups |
| 12. Lachmann et al.  (2007)  Austria | Interleukin-1 β (IL-1β), Plasminogen activator inhibitor type 2 (PAI-2), Prostaglandin E2 (PGE_2_) | Unclear diagnosis of healthy and peri-implantitis groups, lack of valid data (mean, SD) |
| 13. Arikan et al.  (2008)  Turkey | Soluble receptor activator of nuclear factor kappa-B ligand (sRANKL) Osteoprotegerin (OPG) | Due to the unbalanced case numbers, there are no statistical comparisons between H (79), M (4) and P (3) Implants |
| 14. Duarte et al.  (2009)  Brasil | Interleukin 4, 10,12 (IL-4, Il-10, IL -12)  Tumor necrosis factor alpha (TNF-α), Receptor activator of nuclear factor kappa-B ligand (RANKL), Osteoprotegerin (OPG) | Lack of valid data (mean, SD) |
| 15. Luo et al.  (2011)  China | High mobility group box 1 (HMGB1), High mobility group nucleosomal binding domain 2 (HMGN2), Interleukins 1ß, 6, 8 (IL-1ß, IL-6, IL-8),  Tumor necrosis factor alpha (TNF-α) | Healthy implants definition as those having received peri-implant surgery. Same data as in Xie et al. (2011) |
| 16. Xie et al.  (2011)  China | High mobility group box 1 (HMGB1), High mobility group nucleosomal binding domain 2 (HMGN2), Interleukins 1ß, 6, 8 (IL-1ß, IL-6, IL-8), Tumor necrosis factor alpha (TNF-α) | Healthy implants definition as those having received peri-implant surgery. Same data as in Luo et al. (2011) |
| 17. Özçakır-Tomruk et al.  (2012)  Switzerland | Tenascin-C (TNC), matrix metalloproteinase 9 (MMP-9) | Unclear criteria for the diagnosis of healthy and peri-implant disease groups |
| 18. Severino et al.  (2011)  Brazil | Interleukins 6, 8, 10, 17, (IL-6, IL-8, IL-10, IL-17) | Lack of valid data (mean, SD) |
| 19. Hall et al.  (2011)  Sweden | Interleukin-1 β, Tumor necrosis factor alpha (TNF-α), Receptor activator of nuclear factor kappa-B ligand (RANKL), Osteopprotegerin (OPG), Dickkop-related protein-1 (DKK-1), Tartrate-resistent acid phosphatase (TRAP), Cathepsin K (Cat K), Osteocalcin (OC), Alkaline phosphatase (ALP) | Lack of valid data (mean, SD) |
| 20. Özçakır-Tomruk et al.  (2012)  Switzerland | Tenascin-C (TNC), matrix metalloproteinase 9 (MMP-9) | Unclear criteria for the diagnosis of healthy and peri-implant disease groups |
| 21. Arakawa et al.  (2012)  Japan | Matrix metalloproteinases 1, 8, 13 (MMP -1, MMP -8, MMP-13) | Diagnosis of peri-implantitis based exclusively on radiographic measuring active periods of bone loss by annually adjusted vertical bone loss.  Lack of valid data due to limited sample size and the type of assay. |
| 22. Güncü et al.  (2012)  Turkey | Interleukin1β (IL-1β), Interleukin 10 (IL-10), Receptor activator of nuclear factor kappa-B ligand (RANKL), Osteoprotegerin (OPG) | Unclear criteria of the diagnosis of healthy and peri-implant disease groups |
| 23. Yamalik et al.  (2012)  Turkey | Cathepsin K (Cat-K) | Material and methods are close to identical as in Yamalik et al. (2011) Int J Oral Maxillofac Implants 2011; 26:991-997 |
| 24. Rakic  et al.  (2013)  Serbia | Receptor activator of nuclear factor kappa-B (RANK), Soluble receptor activator of nuclear factor kappa-B ligand (sRANKL), Osteoprotegerin (OPG) | The patient population partially overlapped with that of Rakic et al. J Periodontol. 2014 Nov;85(11):1566-74. |
| 25. Rakić et al.  (2013)  Serbia | Receptor activator of nuclear factor kappa-B (RANK) | It is unclear whether the patient population was the same as that of Rakić et al. (2013) Clin. Oral Impl. Res. 24, 2013, 1110–1116. |
| 26. Hall et al.  (2015)  Sweden | Interleukins1ß, 8 (IL-1ß, IL-8), Tissue plasminogen activator (tPA), Plasminogen activator inhibitor 2 (PAI-2), Tartrate-resistant acid phosphatase (TRAP), Cathepsin-K | Evaluation of gene expressions instead of levels of biomarkers |
| 27. Malik et al.  (2015)  India | Myeloperoxidase (MPO), Alkaline Phosphatase (ALP) | Unclear distribution of patients and implants between H, M, and P groups. Lack of differential diagnosis of mucositis and peri-implantitis. |
| 28. Renvert et al.  (2015)  Sweden | Interleukin 1ß (IL-1 ß), Interleukin-1 receptor antagonist (IL-1ra), Interleukins 6, 8, 17, IP-10, MIP-1 α, TNF- α, PDGF, VEGF  . | Lack of a H control group |
| 29. Ramseier et al  (2016)  Switzerland | Interleukin 1ß (IL-1 ß), Matrix  metalloproteinase (MMP)-3, MMP-8, MMP-1, MMP-1 bound to tissue inhibitor of MMP (TIMP)-1  (MMP-1/TIMP-1) | Lack of clear disease conditions |
| 30. Severino et al.  (2016)  Brazil | Interleukin 6, 10, 17, 33, (IL-6, IL-10, IL-17, IL-33) | Lack of valid data (mean, SD) |
| 31. Teixeira et al.  (2017)  Brazil | Interleukin 1β, 4, 6, 10, 17A, 17F, 21, 22, 23, 25, 31, 33 (IL-1β, IL-4, IL-6, IL-10, IL-17A, IL-17F, IL-21, IL-22, IL-23, IL-25, IL-31, IL-33), Interferon-γ (IFN-γ), sCD40L, Tumor necrosis factor α (TNF-α) | Lack of the H control group |
| 32. Che et al.  (2017)  China | Lectin-type oxidized LDL receptor 1 (LOX-1), Interleukin 1 β (IL-1β), Matrix metalloproteinase 2 (MMP-2), Matrix metalloproteinase 9 (MMP-9) | Lack of valid data (mean, SD) |
| 33. Gürlek et al.  (2017)  Turkey | Interleukin 1β, 17A, 17F, 17E (IL-1 β, IL-17A, IL-17F, IL-17E), Soluble receptor activator of nuclear factor kappa-B ligand (sRANKL), Osteoprotegerin (OPG) | Lack of valid data (mean, SD) |
| 34. Che et al.  (2017)  China | Osteopontin (OPN) | Lack of valid data (mean, SD) |
| 35. Sakamoto  et al.  (2018)  Japan | Calprotectin, Cross-linked N-telopeptide of type I collagen (NTx) | Lack of differential diagnosis of mucositis and peri-implantitis. The generic definition "diseased implants" was used. |
| 36. Zhang et al.  (2019)  China | Receptor activator of nuclear factor kappa-B ligand (RANKL) | Lack of valid data (mean, SD) |
| 37. Lira-Junior et a.  (2020)  N/A | Colony-stimulating factor 1 (CSF)-1, Interleukin 1β, 34 (IL-1β, IL-34) | Lack of a H control group |
| 38. Rakic et al.  (2020)  Serbia | Soluble receptor activator of nuclear factor kappa-B ligand (sRANKL), Osteoprotegerin (OPG), RANKL/OPG | Lack of valid data (mean, SD) |
| 39. Zhang et al.  (2020)  China | Matrix metalloproteinase 9 (MMP9), Lectin-type oxidized LDL receptor 1 (LOX-1), Extracellular signal-regulated kinase 1/2) (ERK1/2) | Lack of valid data (mean, SD) |
| 40. Kulkarni et al.  (2020)  India | C-reactive protein (CRP) | Lack of information on study design, differential diagnosis of H and P groups, PICF sample collection and type of assay. |
| 41. Turkoglu et al.  (2020)  Turkey | Cathelicidin LL-37  proteinase 3 (PR3)) | Lack of valid data (mean, SD) |
| 42. Alshiddi et al.  (2021)  Saudi Arabia | Soluble urokinase plasminogen activator receptor (suPAR), Galectin-1, Interleukin 1β (IL-1β) | Lack of differential diagnosis of mucositis and peri-implantitis. The generic definition "peri-implant disease" was used. |
| 43. Kido et al.  (2021)  Japan | Calprotectin | Lack of differential diagnosis of mucositis and peri-implantitis.  Lack of valid data (mean, SD) |
| 44. Sahoo et al.  (2021)  China | Interleukin 1β, 2, 4, 6 (IL-1β, IL-2, IL-4, IL-6), Monocyte chemoattractant protein 1 (MCI-1), Tumor necrosis factor α (TNF-α) | Measurement in the saliva samples. |
| 45. Wang et al  (2022)  China | Interleukin 1β, 6 (IL-1β, IL-6), Tumor necrosis factor α (TNF-α), Sirtuin 1 (SIRT1), C-reactive protein (CRP) | Lack of valid data |
| 46. Kandaswamy et al.  (2022)  USA | Interleukin 1β, 2, 4, 6, 8, 10, 12, 13  **(IL**-1 β, IL-2, IL-4, IL-6, IL-8, IL-10, IL-12, IL-13), Tumor necrosis factor α (TNF-α), Interferon γ (IFN-γ) | Lack of valid data (mean, SD) |
| 47. Djuran et al.  (2023)  Serbia | Receptor activator of nuclear factor kappa-B ligand (RANKL) | Lack of valid data (mean, SD) |
| 48. Rakic et al.  (Epub 2023)  Serbia | Vascular endothelial growth factor (VEGF) | Lack of valid data (mean, SD) |
| 49. Taha  (Epub 2023)  Iraq | Interleukin 10 (IL-10) | Lack of diagnosis of healthy and peri-implantitis |

**References to Table 1 S**

1. Adonogianaki E, Mooney J, Wennström JL, Lekholm U, Kinane DF (1995). Acute-phase proteins and immunoglobulin G against Porphyromonas gingivalis in peri-implant crevicular fluid: a comparison with gingival crevicular fluid. Clin Oral Implants Res, Mar;6(1):14-23. doi: 10.1034/j.1600-0501.1995.060102.x. PMID: 7545442.
2. Salcetti JM, Moriarty JD, Cooper LF, Smith FW, Collins JG, Socransky SS, Offenbacher S (1997). The clinical, microbial, and host response characteristics of the failing implant. Int J Oral Maxillofac Implants, Jan-Feb;12(1):32-42. PMID: 9048452.
3. Fiorellini JP, Nevins ML, Sekler J, Chung A, Oringer RJ (2000). Correlation of peri-implant health and aspartate aminotransferase levels: a cross-sectional clinical study. Int J Oral Maxillofac Implants, Jul-Aug;15(4):500-4. PMID: 10960982.
4. Nomura T, Ishii A, Shimizu H, Taguchi N, Yoshie H, Kusakari H, Hara K (2000). Tissue inhibitor of metalloproteinases-1, matrix metalloproteinases-1 and -8, and collagenase activity levels in peri-implant crevicular fluid after implantation. Clin Oral Implants Res, Oct;11(5):430-40. doi: 10.1034/j.1600-0501.2000.011005430.x. PMID: 11168235.
5. Murata M, Tatsumi J, Kato Y, Suda S, Nunokawa Y, Kobayashi Y, Takeda H, Araki H, Shin K, Okuda K, Miyata T, Yoshie H (2002). Osteocalcin, deoxypyridinoline and interleukin-1beta in peri-implant crevicular fluid of patients with peri-implantitis. Clin Oral Implants Res, Dec;13(6):637-43. doi: 10.1034/j.1600-0501.2002.130610.x. PMID: 12519339.
6. Ataoglu H, Alptekin NO, Haliloglu S, Gursel M, Ataoglu T, Serpek B, Durmus E (2002). Interleukin-1beta, tumor necrosis factor-alpha levels and neutrophil elastase activity in peri-implant crevicular fluid. Clin Oral Implants Res, Oct;13(5):470-6. doi: 10.1034/j.1600-0501.2002.130505.x. PMID: 12453123.
7. Hultin M, Gustafsson A, Hallström H, Johansson LA, Ekfeldt A, Klinge B (2002). Microbiological findings and host response in patients with peri-implantitis. Clin Oral Implants Res, Aug;13(4):349-58. doi: 10.1034/j.1600-0501.2002.130402.x. PMID: 12175371.
8. Kivelä-Rajamäki M, Maisi P, Srinivas R, Tervahartiala T, Teronen O, Husa V, Salo T, Sorsa T (2003). Levels and molecular forms of MMP-7 (matrilysin-1) and MMP-8 (collagenase-2) in diseased human peri-implant sulcular fluid. J Periodontal Res, Dec;38(6):583-90. doi: 10.1034/j.1600-0765.2003.00688.x. PMID: 14632921.
9. Kivelä-Rajamäki MJ, Teronen OP, Maisi P, Husa V, Tervahartiala TI, Pirilä EM, Salo TA, Mellanen L, Sorsa TA (2003). Laminin-5 gamma2-chain and collagenase-2 (MMP-8) in human peri-implant sulcular fluid. Clin Oral Implants Res, Apr;14(2):158-65. doi: 10.1034/j.1600-0501.2003.140204.x. PMID: 12656874.
10. Liskmann S, Zilmer M, Vihalemm T, Salum O, Fischer K (2004). Correlation of peri-implant health and myeloperoxidase levels: a cross-sectional clinical study. Clin Oral Implants Res, Oct;15(5):546-52. doi: 10.1111/j.1600-0501.2004.01061.x. PMID: 15355396.
11. Monov G, Strbac GD, Baron M, Kandler B, Watzek G, Gruber R (2006). Soluble RANKL in crevicular fluid of dental implants: a pilot study. Clin Implant Dent Relat Res, 8(3):135-41. doi: 10.1111/j.1708-8208.2006.00012.x. PMID: 16919021.
12. Lachmann S, Kimmerle-Müller E, Axmann D, Scheideler L, Weber H, Haas R (2007). Associations between peri-implant crevicular fluid volume, concentrations of crevicular inflammatory mediators, and composite IL-1A -889 and IL-1B +3954 genotype. A cross-sectional study on implant recall patients with and without clinical signs of peri-implantitis. Clin Oral Implants Res, Apr;18(2):212-23. doi: 10.1111/j.1600-0501.2006.01322.x. PMID: 17348886.
13. Arikan F, Buduneli N, Kütükçüler N (2008). Osteoprotegerin levels in peri-implant crevicular fluid. Clin Oral Implants Res, Mar;19(3):283-8. doi: 10.1111/j.1600-0501.2007.01463.x. Epub 2007 Dec 13. PMID: 18081868.
14. Duarte PM, de Mendonça AC, Máximo MB, Santos VR, Bastos MF, Nociti FH (2009). Effect of anti-infective mechanical therapy on clinical parameters and cytokine levels in human peri-implant diseases. J Periodontol, Feb;80(2):234-43. doi: 10.1902/jop.2009.070672. PMID: 19186963.
15. Luo L, Xie P, Gong P, Tang XH, Ding Y, Deng LX (2011). Expression of HMGB1 and HMGN2 in gingival tissues, GCF and PICF of periodontitis patients and peri-implantitis. Arch Oral Biol, Oct;56(10):1106-11. doi: 10.1016/j.archoralbio.2011.03.020. Epub 2011 May 12. PMID: 21570059.
16. Xie P, Deng LX, Gong P, Ding Y, Tang XH (2011). Expression of HMGB1 and HMGN2 in gingival tissues, GCF and PICF of periodontitis patients and peri-implantitis. Braz J Microbiol, Jul;42(3):1213-9. doi: 10.1590/S1517-838220110003000047. Epub 2011 Sep 1. PMID: 24031744; PMCID: PMC3768754.
17. Özçakır-Tomruk C, Chiquet M, Mericske-Stern R (2012). Tenascin-C and matrix metalloproteinase-9 levels in crevicular fluid of teeth and implants. Clin Implant Dent Relat Res, Oct;14(5):672-81. doi: 10.1111/j.1708-8208.2010.00319.x. Epub 2011 Mar 23. PMID: 21429069.
18. Severino VO, Napimoga MH, de Lima Pereira SA (2011). Expression of IL-6, IL-10, IL-17 and IL-8 in the peri-implant crevicular fluid of patients with peri-implantitis. Arch Oral Biol, Aug;56(8):823-8. doi: 10.1016/j.archoralbio.2011.01.006. Epub 2011 Feb 8. PMID: 21306703.
19. Hall J, Britse AO, Jemt T, Friberg B (2011). A controlled clinical exploratory study on genetic markers for peri-implantitis. Eur J Oral Implantol, Winter;4(4):371-82. PMID: 22282733.
20. Özçakır-Tomruk C, Chiquet M, Mericske-Stern R (2012). Tenascin-C and matrix metalloproteinase-9 levels in crevicular fluid of teeth and implants. Clin Implant Dent Relat Res, Oct;14(5):672-81. doi: 10.1111/j.1708-8208.2010.00319.x. Epub 2011 Mar 23. PMID: 21429069.
21. Arakawa H, Uehara J, Hara ES, Sonoyama W, Kimura A, Kanyama M, Matsuka Y, Kuboki T (2012). Matrix metalloproteinase-8 is the major potential collagenase in active peri-implantitis. J Prosthodont Res, Oct;56(4):249-55. doi: 10.1016/j.jpor.2012.07.002. Epub 2012 Oct 18. PMID: 23083963.
22. Güncü GN, Akman AC, Günday S, Yamalık N, Berker E (2012). Effect of inflammation on cytokine levels and bone remodelling markers in peri-implant sulcus fluid: a preliminary report. Cytokine, Aug;59(2):313-6. doi: 10.1016/j.cyto.2012.04.024. Epub 2012 May 14. PMID: 22592038.
23. Yamalik N, Günday S, Uysal S, Kilinç K, Karabulut E, Tözüm TF (2012). Analysis of cathepsin-K activity at tooth and dental implant sites and the potential of this enzyme in reflecting alveolar bone loss. J Periodontol, Apr;83(4):498-505. doi: 10.1902/jop.2011.110232. Epub 2011 Aug 26. PMID: 21870974.
24. Rakic M, Lekovic V, Nikolic-Jakoba N, Vojvodic D, Petkovic-Curcin A, Sanz M (2013). Bone loss biomarkers associated with peri-implantitis. A cross-sectional study. Clin Oral Implants Res, Oct;24(10):1110-6. doi: 10.1111/j.1600-0501.2012.02518.x. Epub 2012 Jun 18. PMID: 22708989.
25. Rakić M, Nikolić-Jakoba N, Struillout X, Petković-Curcin A, Stamatović N, Matić S, Janković S, Aleksić Z, Vasilić D, Leković V, Vojvodić D (2013). Receptor activator of nuclear factor kappa B (RANK) as a determinant of peri-implantitis. Vojnosanit Pregl, Apr;70(4):346-51. doi: 10.2298/vsp1304346r. PMID: 23700937.
26. Hall J, Pehrson NG, Ekestubbe A, Jemt T, Friberg B (2015). A controlled, cross-sectional exploratory study on markers for the plasminogen system and inflammation in crevicular fluid samples from healthy, mucositis and peri-implantitis sites. Eur J Oral Implantol, Summer;8(2):153-66. PMID: 26021226.
27. Malik N, Naik D, Uppoor A (2015). Levels of Myeloperoxidase and Alkaline Phosphatase in Periimplant Sulcus Fluid in Health and Disease and After Nonsurgical Therapy. Implant Dent, Aug;24(4):434-40. doi: 10.1097/ID.0000000000000277. PMID: 25996788.
28. Renvert S, Widén C, Persson GR (2015). Cytokine expression in peri-implant crevicular fluid in relation to bacterial presence. J Clin Periodontol, Jul;42(7):697-702. doi: 10.1111/jcpe.12422. PMID: 26085219.
29. Ramseier CA, Eick S, Brönnimann C, Buser D, Brägger U, Salvi GE (2016). Host-derived biomarkers at teeth and implants in partially edentulous patients. A 10-year retrospective study. Clin Oral Implants Res, Feb;27(2):211-7. doi: 10.1111/clr.12566. Epub 2015 Feb 16. PMID: 25682848.
30. Severino VO, Beghini M, de Araújo MF, de Melo MLR, Miguel CB, Rodrigues WF, de Lima Pereira SA (2016). Expression of IL-6, IL-10, IL-17 and IL-33 in the peri-implant crevicular fluid of patients with peri-implant mucositis and peri-implantitis. Arch Oral Biol, Dec;72:194-199. doi: 10.1016/j.archoralbio.2016.08.021. Epub 2016 Aug 24. PMID: 27608364.
31. Teixeira MKS, Lira-Junior R, Telles DM, Lourenço EJV, Figueredo CM (2017). Th17-related cytokines in mucositis: is there any difference between peri-implantitis and periodontitis patients? Clin Oral Implants Res, Jul;28(7):816-822. doi: 10.1111/clr.12886. Epub 2016 Jun 9. PMID: 27283128.
32. Che C, Liu J, Ma L, Xu H, Bai N, Zhang Q (2017). LOX-1 is involved in IL-1β production and extracellular matrix breakdown in dental peri-implantitis. Int Immunopharmacol, Nov;52:127-135. doi: 10.1016/j.intimp.2017.09.003. Epub 2017 Oct 12. PMID: 28898769.
33. Gürlek Ö, Gümüş P, Nile CJ, Lappin DF, Buduneli N (2017). Biomarkers and Bacteria Around Implants and Natural Teeth in the Same Individuals. J Periodontol, Aug;88(8):752-761. doi: 10.1902/jop.2017.160751. Epub 2017 Apr 25. PMID: 28440740.
34. Che C, Liu J, Yang J, Ma L, Bai N, Zhang Q (2018). Osteopontin is essential for IL-1β production and apoptosis in peri-implantitis. Clin Implant Dent Relat Res, Jun;20(3):384-392. doi: 10.1111/cid.12592. Epub 2018 Feb 15. PMID: 29446213.
35. Sakamoto E, Kido R, Tomotake Y, Naitou Y, Ishida Y, Kido JI (2018). Calprotectin and cross-linked N-telopeptides of type I collagen levels in crevicular fluid from implant sites with peri-implant diseases: a pilot study. Int J Implant Dent, Sep 13;4(1):26. doi: 10.1186/s40729-018-0138-2. PMID: 30209708; PMCID: PMC6135732.
36. Zhang Q, Liu J, Ma L, Bai N, Xu H (2019). LOX-1 is involved in TLR2 induced RANKL regulation in peri-implantitis. Int Immunopharmacol, Dec;77:105956. doi: 10.1016/j.intimp.2019.105956. Epub 2019 Oct 23. PMID: 31655342.
37. Lira-Junior R, Teixeira MKS, Lourenço EJV, Telles DM, Figueredo CM, Boström EA (2020). CSF-1 and IL-34 levels in peri-implant crevicular fluid and saliva from patients having peri-implant diseases. Clin Oral Investig, Jan;24(1):309-315. doi: 10.1007/s00784-019-02935-8. Epub 2019 May 17. PMID: 31102043.
38. Rakic M, Monje A, Radovanovic S, Petkovic-Curcin A, Vojvodic D, Tatic Z (2020). Is the personalized approach the key to improve clinical diagnosis of peri-implant conditions? The role of bone markers. J Periodontol, Jul;91(7):859-869. doi: 10.1002/JPER.19-0283. Epub 2020 Jan 20. PMID: 31773730.
39. Zhang Q, Xu H, Bai N, Tan F, Xu H, Liu J (2020). Matrix Metalloproteinase 9 is Regulated by LOX-1 and erk1/2 Pathway in Dental Peri-Implantitis. Curr Pharm Biotechnol, 21(9):862-871. doi: 10.2174/1389201021666200221121139. PMID: 32081107.
40. Kulkarni S, Oswal P, Kulkarni M, Sawant S, Vas A, Rajguru K (2020). Evaluation of levels of CRP in patients with peri-implantitis: A clinical study. Journal of Advanced Medical and Dental Sciences Research, May, vol.8, issue 5:116-8. doi:10.21276/jamdsr.
41. Turkoglu O, Efeoglu C, Atmaca H (2020). Does peri-implant bone loss affect the LL-37 and proteinase 3 levels in peri-implant sulcus fluid? Int J Implant Dent, Aug 4;6(1):45. doi: 10.1186/s40729-020-00240-8. PMID: 32748292; PMCID: PMC7398999
42. Alshiddi IF, AlMubarak AM, Alqutub MN, Alqarawi FK, Alshahrani FA, Javed F, Vohra F, Abduljabbar T (2021). Peri-implant Sulcular Fluid Galectin-1, Soluble Urokinase Plasminogen Activator Receptor and IL-1β Levels under Peri-implant Inflammatory Conditions. Oral Health Prev Dent, Jan 7;19(1):503-510. doi: 10.3290/j.ohpd.b2082081. PMID: 34585876.
43. Kido R, Kido JI, Nishikawa Y, Sakamoto E, Tomotake Y, Yumoto H (2021). Diagnosis of inflammatory peri-implant diseases using an immunochromatographic assay for calprotectin in peri-implant crevicular fluid. Int J Implant Dent, Oct 8;7(1):106. doi: 10.1186/s40729-021-00386-z. PMID: 34623545; PMCID: PMC8498755.
44. Sahoo SK, Jalaluddin M, Bhuyan L, Dash KC, Mishra S, Mishra P (20219. Assessment of Cytokine and Herpesvirus Level in Peri-implantitis and Healthy Patients. J Pharm Bioallied Sci, Nov;13(Suppl 2):S1418-S1421. doi: 10.4103/jpbs.jpbs_233_21. Epub 2021 Nov 10. PMID: 35018002; PMCID: PMC8687024.
45. Wang Z (2022). Peri-implant crevicular fluid SIRT1 levels decrease in patients with peri-implant inflammatory: A prospective observational study. Transpl Immunol, Oct;74:101659. doi: 10.1016/j.trim.2022.101659. Epub 2022 Jun 30. PMID: 35781023.
46. Kandaswamy E, Sakulpaptong W, Guo X, Ni A, Powell HM, Tatakis DN, Leblebicioglu B (2022). Titanium as a Possible Modifier of Inflammation Around Dental Implants. Int J Oral Maxillofac Implants, Mar-Apr;37(2):381-390. doi: 10.11607/jomi.9271. PMID: 35476868.
47. Djuran B, Tatic Z, Perunovic N, Pejcic N, Vukovic J, Petkovic-Curcin A, Vojvodic D, Rakic M (2022). Underdiagnosis in Background of Emerging Public Health Challenges Related to Peri-Implant Diseases: An Interventional Split-Mouth Study. Int J Environ Res Public Health, Dec 28;20(1):477. doi: 10.3390/ijerph20010477. PMID: 36612810; PMCID: PMC9819861.
48. Rakic M, Canullo L, Radovanovic S, Tatic Z, Radunovic M, Souedain A, Weiss P, Struillou X, Vojvodic D (2024). Diagnostic value of VEGF in peri-implantitis and its correlation with titanium particles: A controlled clinical study. Dent Mater, Jan;40(1):28-36. doi: 10.1016/j.dental.2023.10.003. Epub 2023 Oct 19. PMID: 37865576.
49. Taha GI (2023). Involvement of IL-10 gene polymorphism (rs1800896) and IL-10 level in the development of periimplantitis. Minerva Dent Oral Sci, Nov 30. doi: 10.23736/S2724-6329.23.04844-1. Epub ahead of print. PMID: 38037697.
