# Supplementary material for: Biomarkers in Peri‐Implant Crevicular Fluid of Healthy Implants and Those With Peri‐Implant Diseases: A Systematic Review and Meta‐Analysis
Source: J Oral Pathol Med. 2025 Mar 18;54(5):267–82. doi: 10.1111/jop.13612 (PMC12077954; doi:10.1111/jop.13612)
Supplement: Supplementary file 3 — Table S3. Main characteristics of the selected studies. [file JOP-54-267-s003.docx]

**Supplementary Table 3** - Main characteristics of the selected studies.

| **Authors (year)**  **Country** | **Study**  **design** | **Patients (Pa)**  **F/M**  **Age years** | **Implants/sites**  **F/M**  **Age years** | **Functional loading time** | **Definitions**  **(H/ M/P)** | **Biomarkers** | **PICF sample collection and Type of assay** | **Biomarkers**  **Mean/ Median value** | **Main results** |
| --- | --- | --- | --- | --- | --- | --- | --- | --- | --- |
| Kao  et al. ^24^  (1995)  USA | C-s | **Pa** 12  7 F/5M  Age 33-64  Mean: 49.6 | **H** 12  **P** 12 | 37-87 m | **P:** pain, sup, PD≥2mm rapidly increasing over 6 m, BL, loss of resonation with tapping. | IL-1ß | Periopapers strips for 2min or until the maximum sampling volume  (0.1 μL)  ELISA | **H:** 120.4±73.4pg/μL  **P**: 385.95±209pg/μL | **IL-1ß** level was 320% higher in P than in H mplants  (p< .05) |
| Panagakos  et al. ^25^  (1996)  USA | C-s | **Pa (H)** 5  **Pa (P)** 8  F/M NA  Age NA | **H** 17  **P-e** 27  **P- a** 6 | 7-38 m | **H**: minimal or no gingival inflammation, no localized erythema, no BoP, PD≤4mm,  CAL<2 mm, no BL  **P-e**: gingival inflammation, BoP,  PD≤6mm,  no CAL>2 mm BL≤30%  **P-a**: Bop, interproximal PD>6 mm, CAL>6mm, BL>30% | IL-1ß  TNF-α  Pro IL-1ß | Periopaper strips inserted for 30s into 4 sites (mesial distal, buccal and oral) of each mplant.  Fluid amount evaluated with  Periotron 6000  ELISA | **IL-1ß (pg/site)**  H: 59.47±15.55  (68 sites)  P-e: 460.77±35.67  (108 sites)  P-a: 191.1±21.60  (24 sites)  **Pro IL-1ß pg/site**  P-e: 233.13±14.60  (108 sites)  **TNF-α**  Not detected | **IL-1ß and pro-IL-1ß** levels were very low in H than in P implants ad higher in P-e than in P-a implants.  **TNF-α** was not detected in any samples |
| Aboyoussef  et al. ^26^  (1998)  USA | C-s | **Pa** 29  F/M NA  Age NA | **H** 37  **P-e** 37 | ≥6 m | **H:** no gingival inflammation, no localized erythema or BoP, PD≤4mm, CAL≤2mm, no BL  **P-e**: gingival inflammation, BoP,  PD≤6mm, CAL≤2mm, BL ≤ 30%, no mobility | IL-1ß  PGE_2_  MMP_s_ | Periopaper strips randomly inserted  for 30s into one of 4 possible sites (mesial, distal, buccal, oral) of each implant.  **IL-1ß**  ELISA  **PGE_2_**  EIA Kit  **MMPs**  Gel zymography | **IL-1β (pg/site)**  H: 13.0 ± 5.2  P-e: 609.5 ± 54.3  **PGE_2_ (pg/site)**  H: 986.0±68.8  P-e: 1085.9 ± 79.3  **MMPs**  Lack of valid data  (mean, SD) | **IL-1ß** increased by sixfold in P-e versus H implants.  **PGE_2_** no significant difference in H and P-e implants  No correlation  between **PGE_2_ and IL-1ß levels**  in the same samples |
| Paolantonio  et al. ^27^  (2000)  Italy | C-s | **Pa** 81  46F/35M  Age 31-77 | **H** 27  **M** 27  **P** 27 | ≥30 m | **H**: no supragingival plaque, mBI=0, no signs of mucositis, PD≤3mm, BL≤2mm  **M**: supragingival plaque, mBI=2 or 3, peri-implant mucosa redness and swelling PD≤3mm, BL≤ 2mm  **P**: supragingival plaque, mBI=2 or 3, Sup, PD ≥6 mm at least 1 site, BL≥50% of the implant length, mobility | AST | Sterile endodontic paper point (#40) inserted for 30 s into the deepest PD site in P implants or randomly into the mesial or distal site of H and M implants.  Fluid amount evaluated by analytical balance.  Spectrophotometricassay at 25°C | **Mean±SD (U/mL)**  **H**: 0.26±0.16  **M**: 0.38±0.27  **P**: 0.62±0.29  **Median (U/mL)**  **H**: 0.22 (0.06-0.79)  **M**: 0.34 (0.04-1.19)  **P**: 0.55 (0.25-1.34) | Statistically significant difference (p<0.01) in **AST activity** between H, M and P implants, H and P implants, and M and P implants. No significant difference (p>0.1) between H and M implants.  A significant association  (p<0.01) between AST, PD (r=0.55), BL (r=0.60) and Bop (r=0.67). |
| Plagnat  et al. ^28^  (2002)  Switzerland | C-s | **Pa (H**) 7  6F/1M  Age 27-63  **Pa (P**) 8  4F/4M  Age 36-65 | **H** 11  **P** 11 | **H** 27-99 m  **P** 6-84 m | **H**: PI<1, GI<1, PD≤4mm, no Sup, no BL  **P**: BoP and/or Sup, PD>5mm at least one site, BL>20% at least one site (mesial or distal) | EA  α2-M  ALP | Periopaper strips inserted for 15s into 2 sites (mesial and distal) of each implant.  Fluid amount evaluated with  Periotron 8000.  **EA** Fluorogenic substrate  **α2M**  ELISA  **ALP**  p-nitrophe  nyl-phosphate as substrate | The results were reported as total amounts per 15-second samples. Sites with levels below the detectability limit were scored as 0 ng.  **EA (ng/sample**)  H: 1.8±1.2  P: 23.1±12.6  **α2M (ng/sample) ***  H: 3.1±1.1  P: 25.2±14.6  **ALP (U/sample)**  H: 24.1±6.35  P: 142.3±51.1  * a2-macroglobulin was absent from most of the samples belonging to the healthy group | **EA, α2-M** and **ALP** were significantly higher in P than in H implants 13, 8, and 6 times, respectively.  **EA** was correlated with PD and BL, **α2M** with GI, and **ALP** with GI and BL |
| Yalçın  et al. ^29^  (2005)  Turkey | C-s | **Pa (H**) 12  5F/7M  Age  40.90±7.00  **Pa (M**) 13  4F/9M  Age  43.69±7.49 | **H** 24  **M** 24 | 13 m | **H**: no gingival inflammation, GI low scores, PD≤3 mm  **M**: gingival inflammation, GI increasing scores, PD≤5mm | PGE_2_ | Periopaper strips inserted for 30s into one site (mesio-buccal) of each implant.  EIA | **H**: 40.02±4.81  **M**: 58.27±7.94 | **PGE_2_ levels** were statistically significantly higher (p>0.05) in M than in H implants and had a statistically significant positive correlation (p<0.05) with GI and PD but no (p>0.05) with PI.  In the H implants, No statistically significant positive correlation (p>0.05) of **PGE^2^** with clinical parameters |
| Zhang  et al. ^30^  (2005)  China | C-s | **Pa** 56  25F/31M  Age 31.2  (20-55) | **H** 23  **M** 35  **P** 8 | ≥3 m | **H:** no PI, no gingival inflammation, PD<4mm, no BL  **M:** GI>=1, PI>=1, PD>4mm at least one site, no BL  **P:** GI>=1, PI>=1, PD>4mm at least one site, BL>20% at least one site (mesial or distal). | IL-6 | Sterile endodontic paper point (#40) inserted for 30 s into 2 sites (mesial and distal) of each implant.  Fluid amount evaluated by analytical balance.  ELISA | **IL-6 (ng/mL)**  H: 1.74±1.66  M: 5.53±1.57  P: 9.36±5.56 | **Il-6** was significantly lower in H than in M (p<0.05) and P (p<0.01) implants and in M than P implants (p<0.01) |
| Paknejad  et al. ^31^  (2006)  Iran | C-s | **Pa (H**) 13  5F/8M  Age  40.90±7.00  **Pa (P**) 12  4F/8M  Age 43.69±7.49 | **H** 17  **P** 17 | 41 m | **H**: no P, no BoP, no signs of M, BL≤⅓ length of the first step or second thread of implant.  **P**: PI, Bop, BL>⅔ length of the first step of implant or exposure of more than 2 threads | AST  ALP | Periopaper strips inserted for 30s into the sulcus/  pocket of each implant.  **AST**  **activity**  p-Nitrophenyl Phosphate  Substrate (pNPP)/ ELISA  **AL**  **Pactivity**  L-aspartate  :2-oxoglutarate aminotrans=ferase  (EC 2.6.1.1) | **AST activity (mean±SD)**  H: 1.70 ±0.98 IU/mL  P: 6.41±1.09 IU/mL  **ALT activity (mean±SD)**  H: 1.17±1.01 IU/mL  P: 3.05±1.47 IU/mL | **AST and ALP activity** had a significant higher in P than H implants. (p<0.0001)  **AST activity** was significantly associated with BoP (p=0.02)  **ALP activity** had no statistical correlation between increased PI and BoP (p=0.05) |
| Strbac  et al. ^32^  (2006)  Austria | C-s | **Pa** 19  13F/6M  Age 61  (30–84) | **H** 40  **P** 40 | ≥1y | **P:** mPI≥1.00, MBI>1.00, PD>3.00 mm, exposed implant threads visible | Cat-K | Periopaper strips inserted for 30s into 2 sites (buccal and oral) of each  implant.  Fluid amount evaluated with  Periotron 8000.  **Cat K**  Sandwich ELISA  **Total protein**  bicinchoninic method | **Median**  **Cat K absolute amount (pmol/sample)**  H:10.1 (0-33.5)  P: 22.4 (3.7-56.3)  **Cat K concentration**  **(nM/sample)**  H: 2.2 (0.01-6.4)  P: 1.7 (0.4-4.6)  **Cat K normalized to protein (nM/µg)**  H: 0.07 (0–2.4)  P: 0.09 (0.01–2.5) | **Cat K total amount** was higher in P than in H implants**.**  **Cat K levels** and **Cat K concentration** were positively correlated with PD, mPI and mBI. |
| Xu  et al. ^33^  (2008)  USA | C-s | **Pa (H**) 5  4F/1M  Age 50-72  **Pa (P**) 5  3F/2M  Age 50-72 | **H** 16  **P** 7 | ≥ 1 y | **H:** PD<3mm, no BL  **P:** inflamed peri-implant mucosa, BoP, PD 4-6 mm, BL, no mobility | MMP-8 | Periopaper strips inserted for 10s into the mesial-buccal site of each implant.  Fluid amount evaluated with Peritron 6000.  **MMP-8 activity**  DNP-synthetic octapeptide  **MMP-8 concentation**  Western immunoblot analysis | **MMP-8 activity/site**  H: 0.4±0.1  P: 14.4±3.8  **MMP-8 activity/µL**  H: 2.1±0.5  P: 22.5 ±4.8 | **MMP-8 activity** per site and µL was significantly higher in the P than in H implants (p<0.05) |
| Tümer  et al. ^34^  (2008)  Turkey | C-s | **Pa** 15  F/M NA  Age 43.5 | **H** 15  **P** 15 | ≥6 m | **H:** no BL  **P:** inflammatory process with at least three threads of BL | ICTP  Osteocalcin | Periopaper strips inserted for 30s into 4 sites (mesial, distal, buccal, lingual/palatal) of each implant.  Fluid amount evaluated with  Periotron 8000.  Radioimmunoassay | **ICTP concentration (ng/mL^-1^)**  H: 12.435±10.750  P: 13.178±6.827  **Osteocalcin concentration (ng/mL^-1^)**  H: 4.054±2.430  P: 6.009±5.206 | **ICTP** had no statistically significant difference between P and H implants  **Osteocalcin** had a significant increase (p< 0.05)  between P and H implants. |
| Petković  et al. ^35^  (2010)  Serbia | C-s | **Pa (H)** 49  **Pa (M-e)** 30  **Pa (M-a)** 11  9F/81M  Age 55 | NA | 12-36 m | **H**: normal gingiva (GI=0)  **M-e**: mild inflammation, a slight change in colour, slight oedema, but no BoP (GI=1)  **M-a**: moderate inflammation, redness, oedema  and glazing, BoP (GI=2)  **P**: severe inflammation with marked redness and oedema, ulceration, tendency for spontaneous bleeding (GI=3). | IL-1ß  TNF-α  IL-8  MIP-1α | Periopaper strips inserted for 30s into the sulcus/  pocket of each implant.  Fluid amount evaluated with  Periotron 6000.  ELISA | **IL-1β (pg/mL)**  H: 12.6±14.8  M-e: 178.7±208.1  M-a: 1705.3±2410.6  **TNF-α** **(pg/mL)**  H: 0.8±1.85  M-e: 19.8±23.6  M-a: 130.4±74.1  **IL-8 (pg/mL)**  H: 47.5±29.3  M-e: 08.8±182.5  M-a: 1659.0±1506.8  **MIP-1 α (pg/mL)**  H: 8.2±8.3  M-e: 61.8±28.3  M-a: 150.5±50.1 | **IL-1ß** (p<0.01)**, TNF-α** (p<0.001)**, IL-8** (p<0.001) and **MIP-1α** (p<0.001) levels were significantly lower in H than in M-e and M-a implants  and significantly higher in M-a than in M-e  implants |
| Mierzwinska-Nastalska  et al. ^36^  (2010)  Poland | C-s | **Pa** 30  17F/13M  Age 58.5  (35-82) | **H** 20  **P** 39 | ≥ 1y | **H:** no PI, no BoP, no gingival erythema, PD≤2mm  **P**: swelling and gingival erythema, BoP, PD>3mm, BL | VEGF | Sample syringe with a blunt needle inserted into the sulcus/  pocket of each implant to collect 0.3 mL.  ELISA | **VEGF (pg/mL)**  H: 25.21 ±0.32  P: 86.28 ±0.21 | **VEGF** level was significantly higher in P than in H implants (p<0.001) and strongly correlated with PI and PD |
| Sarlati  et al. ^37^  (2010)  Iran | Analytical study | **Pa** NA | **H** 13  15F/11M  Age 49.92±1  **M** 14  20F/8M  Age 52.21±  1.22  **P** 13  19F/7M  Age 3.59±  0.94 | ≥ 6 m | **H:** No PI and calculus, no BoP, no Sup, PD≤3mm, no BL  **M**: BoP, no Sup, PD≤5mm, no BL  **PI:** BoP and/or Sup, PD>5mm, BL | sRANKL | Periopaper strips inserted for 30 s into 2 sites (buccal and oral) of each implant.  ELISA | **sRANKL (pmol/l)**  H: 0.2892±0.01  M: 0.2851±0.006  P: 0.2876±0.006 | **sRANKL levels** had no statistically significant difference between H, M, and P groups (p=0.12) |
| Arikan  et al. ^38^  (2011)  Turkey | C-c | **Pa** 28  13F/15M  **Pa(H)** 16  Age: 52±11  **Pa(P)** 12  Age: 56±14 | **H** 21  **P** 18 | **H** 3.0±0.9 y  **P** 4.0±0.7 y | **H**: no PI, no BoP, no Sup, no gingival inflammation, PD≤4mm, no BL  **P**: BoP and/or Sup, PD≥5mm at least one site, BL at least 3 threads in at least one site but no more than half of the implant length | ICTP  sRANKL  OPG | Periopaper strips inserted for 30 s into 2 sites (buccal and lingual) of each implant.  Fluid amount evaluated with  Periotron 8000.  ELISA | **ICTP concentration**  (**pg/µL)**  H: 1.6±0.2  P: 1.7±1.1  **ICTP total amount (pg/2 samples)**  H: 0.8±0.2  P: 1.6±0.2  **sRANKL concentration**  (**pg/µL)**  H: 21.8±8.1  P: 16.6±7.2  **sRANKL total amount (pg/2 samples)**  H: 10.9±3.0  P: 10.0±22  **OPG concentration**  (**pg/µL)**  H: 57.9±16.2  P: 14.5±15.0  **OPG total amount (pg/2 samples)**  H: 25.5±0.4  P: 11.9± 4.9  **sRANKL/OPG**  H: 0.4±0.2  P: 0.8±0.9 | **ICTP total amount** was significantly higher in P than in H implants (p<0.001).  **sRANKL concentration**  was significantly higher in H than in P implants  (p<0.005)  **OPG total amount and OPG concentration** was significantly higher in H than in P implants  (p<0.001).  **sRANKL/OPG**  was not significative |
| Yamalik  et al. ^39^  (2011)  Turkey | C-s | **Pa** 47  32F/15M  Age: 41.50  (20-65) | **H** 27  **M** 26  **P** 15 | NA | **H**: no BL  **M**: no BL  **P**: BL | Cat-K | Periopaper strips inserted for 30 s into 4 sites (mesial, distal, buccal, oral) of each implant. Fluid amount evaluated with  Periotron 8000.  Fluorescence (Ex/Em  400/505) | **Cat-K enzyme activity (units)**  H: 3.440  (min. 0.227-max 16.716)  M: 4.745  (min 0.312-max. 23.529)  P: 10.260  (min. 0.336-max. 28.046) | Significantly higher **Cat K activity** in P than in H and M implants (p=0.0001) |
| Melo  et al. ^40^  (2012)  Brazil | C-s | **Pa (H**) 31  22F/9M  Age: 47.83  (26-71)  **Pa (P**)16  9F/7M  Age: 44.13  (27-66) | **H** 31  **P** 16 | ≥ 1y | **H:** No bleeding, no BoP and/or Sup, PD≤4mm  **P**: N/A | IL-1ß  IL-6 | Periopaper strips inserted  for 30s into the greatest PD or mesio-buccal site of each implant.  Fluid amount evaluated with  Periotron 6000.  ELISA | **IL-1ß (pg/mL)**  H: 2.04±2.74  P: 3.88±5.82  **IL-6 (pg/mL)**  H: 0.32±0.59  P: 0.35±0.48 | No differences in **IL-1ß and IL-6**  levels between H and P implants  (p > 0.05) |
| Darabi  et al. ^41^  (2013)  Iran | C-c | **Pa (H)** 18  11F/7M  Age 40.8±8.6  **Pa (P**) 24  14F/10M  Age 44.4±6.6 | NA | 1y | NA | IL-17  TNF-α | Endodontic paper points (#30) inserted for 10s into the deepest pocket of each implant 4 times at three-minute intervals.  If more than one site had a similar depth, sampling was performed at 6 sites (mesio-buccal, mid-buccal, disto-buccal, mesio-  lingual,  mid-lingual and disto-lingual/palatal).  ELISA | **IL-17 (pg/site)**  H: 14.5 ± 8.9  P: 19.7 ± 16.0  **TNF-α (pg/site)**  H: 14.5 ± 8.9  P: 38.9 ± 9,3 | Significantly higher levels of **IL-17** (p=0.016) and **TNF-α** (p=0.000) in P than in H implants.  No correlation between **IL-17** and **TNF-α** (p=0.331) |
| Casado  et al. ^42^  (2013)  Brazil | C-s | **Pa (H**) 10  7F/3M  Age 49.5  **Pa (M**)10  5F/5M  Age 52.8  **Pa (P**)10  6F/4M  Age 57.4 | **H** 10  **M** 10  **P** 10 | NA | **H**: no clinical signs of inflammation in the peri-implant mucosa, no BL  **M**: BoP, red mucosa and swelling, spontaneous bleeding, no BL  **P**: clinical signs of inflammation, including implant  mobility and suppuration in some cases, BL. | IL-1ß  IL-10 | Calibrated volumetric microcapillary pipettes into the sulcus/  pocket of each implant  Fluid amount evaluated 1µL.  ELISA | **IL-1β (pg/mL)**  H: 67.51 ± 62.9  M: 325.89±235.17  P: 439.89± 182.67  **IL-10 (pg/mL)**  H: 3720± 1237.97  M: 2711.21±866.65  P: 1707.79±948.10 | The mean of **IL-1 ß** levels was significantly lower in H than in the M (p<0.0005) and P (p<0.001) implants.  No significant difference occurred between M and P implants (p>0.05).  **IL-10** levels were significantly higher in H than in M and P implants (p=0.001), and in in M than in P implants (p=0.047). |
| Ata-Ali  et al. ^43^  (2013)  Spain | C-s | **Pa (H)** 22  13F/9M  Age  63.6±10.4  **Pa (M)** 12  7F/5M  Age 60.2±7.4 | **H** 54  **M** 23 | NA | **H**: no clinical signs of inflammation of the peri-implant mucosa, PD<4mm, no BL  **M**: gingival redness, swelling, BoP, no BL. | IL-1ß  IL-6 | Periopaper strips inserted for 30s into the sulcus/  pocket of each mplant.  Fluid amount evaluated with  Periotron 8000.  Cytometric Bead Array (CBA) system and Fluorescence Activated Cell Sorting (FACS) analysis | \| **IL-1ß (pg/mL)**  H: 21.2±NA  M: 42.5±NA  **IL-6 (pg/mL)**  H: 0.53±0.63  M: 0.99±0.49 \| \| --- \| | **IL-1ß** was increased without significant difference in M than in H implants.  **IL-6** expression was significantly greater in M than in H implants (p<0.05) |
| Moura  et al. ^44^  (2013)  Brazil | C-s | **Pa (H)** 10  **Pa(M**) 17  F/M NA  Age NA | **H** 10  **M** 17 | ≥ 1y | **H**: no BoP and PD≤3mm  **M**: BoP and PD>3mm at least one site. | IgA1 | Endodontic paper points (#30) inserted for 30s into 6 sites (mesio-buccal, mid-buccal, disto-buccal, mesio-lingual,  mid-lingual and disto-lingual/palatal) of each implant.  ELISA | **IgA1**  H: 26.6±20.7  M: 25.1±20.5 | No statistically significant difference (p=0.960) of IgA1 levels in H and M implants |
| Rakić  et al. ^45^  (2014)  Serbia | C-s | **Pa (H)** 58  F/M NA  Age  54.66±12.24  (50.00–59.3)  **Pa (M)** 54  F/M NA  Age 51.83±14.94  (36.15–7.52)  **Pa (P)** 52  F/M NA  Age 57.39±13.14  (51.71–3.08) | **H** 58  **M** 54  **P** 52 | **H** 4.84±2.53y (3.86-5.85)  **M** 4±3.56 y (1.29- 8.71)  **P**  6.85±4.59 y  (4.7-8.99) | **H**: no BoP, PD≤3mm. no BL  **M**: BoP, PD≥3mm, no BL compared to Rx at the time of prosthetic replacement  **P**: BoP, PD5≥mm,  BL≥2 implant threads compared to Rx at the time of prosthetic replacement | sRANKL  RANK  OPG  Cat-K  Sclerostin | Periopaper strips inserted for 30s into the mesial site of each implant.  Fluid amount evaluated with  Periotron 6000.  ELISA | **sRANKL (pg/mlL**  H: 5.17±3.47  M: 8.29±13.27  P: 9.25±15.45  **RANK (pg/mL)**  H: 432.19±306.66  M: 992.15±741.46  P: 1286.57±581.11  **OPG (pg/mL)**  H: 15.94±9.11  M: 14.46±1.84  P: 19.02±9.52  **sRANKL/OPG**  H: 0.72±0.63  M: 0.92±1.32  P: 1.01±1.23  **Cat-K (pg/mL)**  H: 439.39±46.03  M: 966.86±44.11  P: 1020.91±372.23  **Sclerostin(pg/mL)**  H: 146.16±95.83  M: 31±4.24  P: 26025.50±3669.19 | **RANK** (P=0.002), **sRANKL** (p=0.01), **OPG** (p=0.03) and **sclerostin**  (p<0.001) were significantly increased in P than in H implants.  **RANK**(p=0.02) and **Cat-K** (p=0.04) were significantly increased in M than in H implants.  **Sclerostin**  (p <0.001) was significantly higher in P than in M implants.  **sRANKL/OPG** **relative ratio** had no significant differences between the three groups. |
| Yaghobee  et al. ^46^  (2014)  Iran | C-s | **Pa** 8  F4/M4  Age NA | **H** 8  **P** 8 | ≥ 1y | **H:** no BoP, no PD>3 mm, pink and stippling gingiva, no exposure of implant threads in Rx  **P**: BoP, PD>5 mm, exposure at least of 2 implant threads in Rx | IL-1ß  IL-6 | Endodontic paper points (#30) inserted for 30s into the deepest sulcus/  pocket of each implant.  ELISA | **IL-1ß**  H: 13.55±8.59  P: 20.76±6.86  **IL-6**  H: 6.43±5.03  P: 8.87±5.84 | **IL-1β** (p=0.002) and **IL-6** (p=0.049) were significantly higher in P than in H implants |
| Ata-Ali  et al. ^47^  (2015)  Spain | C-s | **Pa (H)** 22  13F/9M  Age 63.6±10.4  **Pa (P**) 13  7F/6M  Age 52±7.7 | **H** 54  **P** 24 | ≥ 2y | **H**: no signs of inflammation,  PD <4 mm, no BL  **P**: BoP or Sup,  PD≥4mm, BL, no implant mobility. | IL-1β  IL-6  IL-8  IL-10  TNF-α | Periopaper strips inserted  for 30s into the sulcus/  pocket of each implant  Fluid amount evaluated with  Periotron 8000.  Human Inflammation Cytometric Bead Array (CBA) system  and FACS analysis | **IL-1ß (pg/m)**  H: 21.2±24.2  P: 58.5±84.8  **IL-6**  H: 0.53±0.63  P: 0.96±0.64  **IL-8**  H: 193.9±NA  P: 352±NA  **IL-10**  H: 0.45±0.87  P: 0.91±0.90  **TNF-α**  H: 0.25±0.56  P: 1.08±1.49 | **IL-1β** (p<0.01),  **IL-6** (p<0.01),  **IL-10** (p<0.05) and **TNF-α** (p<0.01) were  significantly  higher in P than H implants  **IL-8** did not increase significantly in P than H sites. |
| Rakić  et al. ^48^  (2015)  Serbia | C-s | **Pa (H)** 189  F90/99M  Age 49.4  (23-60)  **Pa (P)** 180  F78/102M  Age 53.2  (35-66) | **H** 189  **P** 180 | **H** 5.34y  (2-6)  **P** 6.28y  (2-8) | **H**: no signs of inflammation, no BoP, PD≤3mm, no BL  **P**: BoP, PD5≥ mm, BL≥2 implant threads compared to Rx at the time of prosthetic replacement | RANKL  OPG | Periopaper strips inserted for 30s into the sulcus/  pocket of each implant  Fluid amount evaluated with  Periotron 6000.  ELISA | **RANKL (pg/mL)**  H: .15±4.14  P: 8.91±3.43  **OPG (pg/mL)**  H: 11.11±4.39  P: 8.34±4.16  **RANKL/OPG**  H: 0.40±0.33  P: 1.51±1.14 | **RANKL concentration** (p<0.001) and relative ratio **RANKL/OPG** (p<0.001) were significantly higher in the P than in the H implants  **OPG** was significantly higher in the H than in the P implants (p<0.001) |
| Wang  et al. ^49^  (2016)  USA | C-s | **Pa (H)** 34  14F/20M  Age  62.1±10.4  **Pa (P)** 34  19F/15M  Age  65.3±10.3 | **H** 34  **P** 34 | ≥ 6 m | **H**: absence of  radiographic implant threads exposure  **P**: BoP and/or Sup,  PD ≥5 mm, BL with the exposure of the implant surface  surface below the first thread | IL-1ß  MMP-8  OPG  TIMP-2  VEGF | Periopaper strips inserted for 60s into the sulcus/  pocket of each implant.  Array-based  multiplex  ELISA  system | **IL-1ß (pg/ml)**  H: 44.60±53.00  P: 135.83±97.30  **MMP-8 (pg/ml)**  H: 6029.18±2132.07  P: 5943.13±1183.24  **OPG (pg/ml)**  H: 66.51±115.1  P: 111.69±159.00  **TIMP-2** **(pg/ml)**  H: 5488.32±3852.5  P: 9771.82±5113.00  **VEGF (pg/ml)**  H: 59.11±56.7  P: 128.99±121.19 | **IL-1ß** (p<0.001), **TIMP-2** (p=0.001), **VEGF** (p=0.012), and **OPG** (p=0.050) levels increased significantly in the P than in the H implants**.**  **MMP-8 l**evels had no significant difference (p=0.454) among H and P implants |
| Zani  et al. ^50^  (2016)  Brazil | C-s | **Pa (H)** 14  13F/1M  Age 58±7  **Pa (P)** 26  19F/7M  Age 57± 8 | **H** 16  **P** 47 | ≥ 1y | **H**: no BOP or Sup,  PD<5 mm, no BL>2mm  **P**: at least one site with BoP and/or Sup, PD>4mm, BL>2mm. | FGF-2  Eotaxin  Flt-3L  GM-CSF  IL-10  MCP-3  IL-12p40  MDC  IL-12p70  IL-13  PDGF-BB  IL-15  sCD-40L  IL-17  IL-1ra  IL-1 β  IL-2  IL-6  TNFα  VEGF | Periopaper strips inserted for 30s into the mesio-buccal site of each implant.  The human cytokine 20-plex  (magnetic bead panel) Millipore kit  Luminex Multiplay Assay | **FGF-2 (pg/30s)**  H: 1.40±0.64  P: 1.64±0.49  **Eotaxin (pg/30s)**  H: 0.86±0.71  P: 1.24±0.57  **Flt-3L (pg/30s)**  H: 0.84±0.34  P: 0.96±0.34  **GM-CSF (pg/30s)**  H: 0.33±0.42  P: 0.62±0.46  **IL-10 (pg/30s)**  H: 0.87±0.55  P: 1.07±0.49  **MCP-3 (pg/30s)**  H: 0.97±0.49  P: 1.17±0.34  **IL-12p40 (pg/30s)**  H: 0.90±0.52  P: 1.13±0.50  **MDC (pg/30s)**  H: 1.49±0.26  P: 1.72±0.28  **IL-12p70 (pg/30s)**  H: 0.44±0.36  P: 0.67±0.31  **IL-13(pg/30s)**  H: 0.56±0.31  P: 1.79±0.35  **PDGF-BB (pg/30s)**  H: 1.24±0.40  P: 1.63±0.49  **IL-15 (pg/30s)**  H: 0.47±0.35  P: 0.66±0.33  **sCD-40L (pg/30s)**  H: 1.37±0.60  P: 1.80±0.47  **IL-17 (pg/30s)**  H: 0.27±0.27  P: 0.50±0.33  **IL-1ra (pg/30s)**  H: 3.01±0.37  P: 3.28±0.32  **IL-1 β** **(pg/30s)**  H: 1.15±0.91  P: 1.75±0.81  **IL-2 (pg/30s)**  H: 0.37±0.37  P: 0.63±0.40  **IL-6 (pg/30s)**  H: 0.57±0.51  P: 0.97±0.54  **TNF-α (pg/30s)**  H: 0.44±0.35  P: 0.74±0.38  **VEGF (pg/30s)**  H: 1.73±0.55  P: 1.85±0.44 | After adjusting for the false discovery rate, **GM-CSF,** **MDC**, **IL-12p70, IL-13, PDGF-BB, IL-15, sCD40L, IL-17, IL-1 β, IL-2, IL-6 and TNFα** had statistically significant differences between the H and P implants |
| Liu  et al. ^51^  (2017)  China | C-s | **Pa** 47  26F/21M  Age 43±11.7  (18-59) | **H** 39  **M** 24  **P** 16 | 65.2±4.7 m  (57-71) | **H**: PD<3mm, sGI≤1  **M**: PD≥3mm, sGI>1, no BL  **P**: PD≥3mm, GI>1, BL | HMGB1  IL-1β  IL-6  IL-8  TNF-α | Periopaper  strips inserted for 30s into the sulcus/  pocket of each implant  Fluid amount evaluated with  Periotron.  Multiplex  cytokine immunoassay | **HMGB1 (pg/mL)**  H: 167.3±18.9  M: 309.4±45.4  P: 962.2±82.5  **IL-1β (pg/mL)**  H: 227.7±18.5  M: 483.3±65.9  P: 872.2±114.3  **IL-6 (pg/mL)**  H: 144.3±33.1  M: 288.7±45.9  P: 537.2±151.4  **IL-8 (pg/mL)**  H: 45.3±5.9  M: 117.4±16.4  P: 178.26±162.5  **TNF-α (pg/mL)**  H: 178.3±11.9  M: 289.4±7.4  P: 402.6±24.5 | **HMGB1, IL-1β,** **TNF-α** **and IL-8** levels had a significantly increased concentration in the M and P implants than in the H implants and in the P than the M implants  **IL-6** levels were lower in H than in M and P implants |
| Cakal  et al. ^52^  (2018)  Turkey | C-s | **Pa** 111 | **H** 47  34F/13M  Age 51±9  **M** 46  31F/15M  Age 50±10  **P** 52  31F/21M  Age 55±10 | ≥ 6 m | **H**: no BoP, no BL beyond normal remodelling  **M**: BoP, no BL beyond normal remodelling  **P**: BoP at least one implant surface,  PD≥5mm, BL>2mm  from implant platform after initial remodelling | Osteocalcin  Osteopontin  Osteonectin | Periopaper strips inserted for 30s into mesio- buccal site  Fluid amount evaluated by calibrated electronic device.  ELISA | **Osteocalcin (ng/site)**  H: 1.26±0.29  M: 1.22±0.23  P: 1.23±0.27  **Osteopontin (ng/site)**  H: 4.35±0.79  M: 4.26±0.47  P: 4.42±0.63  **Osteonectin (ng/site)**  H: 0.78±0.22  M: 0.79±0.15  P: 0.73±0.25 | No significant differences in **osteocalcin, osteopontin**, and **osteonectin** total amounts between H, M and P implants  (p>0.05) |
| Yakar  et al. ^53^  (2019)  Turkey | C-s | **Pa(H)** 25  12F/13M  Age  50.64±13.36  (22-78)  **Pa (P)** 27  17F/10M  Age  55.85±14.22  (22-76) | **H** 25  **P** 27 | ≥ 6 m | **H:** no sign of inflammation,  no PD<4mm, no BL  **P**: PD≥6mm at  least in one peri-implant site and  at least one between Bop, Sup or BL | Sclerostin  TWEAK  RANKL  OPG | Periopaper strips inserted for 30s into 4 sites (mesio-buccal, mid- buccal, disto-buccal, mid-lingual/  palatal) for each implant  Fluid amount evaluated with  Periotron 8000.  ELISA | **Sclerostin(pg/ml)**  H: 33.39±22.94  P: 63.05±23.62  **TWEAK (pg/ml)**  H: 80.02±89.24  P: 250.35±122.95  **RANKL (pg/ml)**  H: 0.64±0.43  P: 1.84±0.93  **OPG (pg/ml)**  H: 107.87±103.41  P: 190.70±180.02  **RANKL/OPG**  H: 0.0153±0.0171  P: 0.0234±0.0244 | Mean levels of **sclerostin** (p=0.002), **TWEAK** (p<0.0001), **RANKL** (p<0.0001) and **OPG** (p=0.037) were significantly higher in the P than in the H implants |
| Song  et al. ^54^  (2019)  China | C-s | **Pa** 40  16F/24M  Age 38.2±4.3  (24-58) | **H** 10  **P** 42 | ≥ 1y | **H:** SBI≤2, PD≤3mm  **P:** SBI>2, PD>3 mm | IL-6  TNFα  hs-CRP  MMP-8  MMP-13 | Filter paper strips (2 mm × 20 mm) for 1 min.  ELISA | **IL-6 (ng/ml)**  H: 0.61±0.21  P: 4.77±1,29  **TNFα (ng(ml)**  H: 6.01±2.33  P: 19.72±4.53  **hs-CRP (ng/ml)**  H: 5.56±2.383  P: 13.22±5.62  **MMP-8m(mg/L)**  H: 0,12±0.03  P: 3.85±0.45  **MMP-13(mg/L)**  H: 11,32±1.99  P: 17.45±2.28 | **TNF-α, IL-6, hs-CRP, MMP-8** and **MMP-13** levels in P were significantly  higher than in H implants (p<0.01) |
| Sert  et al. ^55^  (2019)  Turkey | C-c | **Pa** 39  0F/39 M  Age  44.61 ± 6.76  (35-55) | **H** 13  **M** 13  **P** 13 | ≥ 1y | **H**: BOP 0%, CAL<2mm, PD<3mm, no BL  **M:** BoP and/or peri-implant mucosa inflammation, CAL<2mm, PD<3mm, no BL  **P:** BoP/Sup, PD>6 mm, attachment or  BL ≥3mm | SP  NKA  CGRP  NPY | Periopaper strips inserted for 30s into the sulcus/  pocket of each implant  Fluid amount evaluated with  Periotron 8000.  ELISA | **SP (pg/µl)**  H: 30.7±2.31  M: 51.05±4.97  P: 135.68±5.80  **NKA (pg/µl)**  H: 66.34±2.77  M: 78.77±4.03  P: 105.59±4.27  **CGRP (pg/µl)**  H: 48.79±1.47 M: 37.29±1.40 P: 23.93±1.80  **NPY (pg/µl)**  H: 585.85±11.19 M: 324.2±25.03 P: 110.03±17,78 | **SP** and **NKA** levels were higher in the M and P implants than in the H implants (p=0.001)  **CGRP** and **NPY** levels decreased in diseased peri-implant sites. |
| Farhad  et al. ^56^  (2019)  Iran | C-c | **Pa (H)**17  10F/7M  Age 49.33±7.89  **Pa (M)** 17  Age 46±7.092  11F/6M  **Pa (P)** 17  8F/9M  Age 49.33±7.89 | NA | ≥ 1 year | **H**: N/A  **M:** inflammation,  PD<4mm, BL to the first thread  **P:** inflammation,  PD>4 mm, BL to the second thread | IL-17  IL-10 | Endodontic paper points (#25) inserted for 4min into 2 sites (the deepest pocket) of each implant.  ELISA | **IL-17 (ng/dl)**  H: 5.8±0.5  M: 57.7±14.6  P: 19.9±10.3  **IL-10 (ng/dl)**  H: 7.7±3.03  M: 38±10.3  P: 56.5±16.4 | **IL‑17** mean value was significantly lower in the H implants than in the M and P implants and in the P than in the M implants (p<0.001)  **IL-10**  mean value was significantly lower in the H implants than in the M and P implants and in the M than in the P implants (p<0.001) |
| Acipinar  et al. ^57^  (2019)  Turkey | C-s | **Pa** 53  **Pa (H)**  9F/7M  Age 44.4±9.1  **Pa (M)**  4F/14M  Age 48.3±10.7  **Pa (P)** 17  7F/12M  Age 54.2±8.2 | **H** 30  **M** 30  **P** 30 | ≥ 1 year | **H**: absence of inflammation, no BL beyond initial remodelling  **M**: presence of inflammation, no BL beyond inizial remodelling  **P**: BoP, and/or Sup, PD≥6mm, BL≥3mm | FGF-23 25(OH)D3 | Paper strips inserted for 30 s into 2 sites (mesial, distal) of each implant  Fluid amount evaluated with  Periotron 8000.  ELISA | **Mean (SE)**  **FGF-23 concentration (pg/mL)**  H: 8.80 (1.47)  M: 8.66 (1.16)  P: 6.90 (1.00)  **Total amount (pg)**  H: 1.81 (1.23)  M: 2.32 (1.37)  P: 2.92 (1.79)  **25(OH)D_3_ concentration (pg/mL)**  H: 10.34 (1.09)  M: 20.21 (1.27)  P: 4.97 (0.64)  **Total amount (pg)**  H: 2.09 (0.40)  M: 1.94 (0.64)  P: 1.74 (0.45) | No statistically significant differences in **FGF-23 concentrations** among implants (p>0 .05)  **25(OH)D3 concentration** was significantly lower in P than in M and H implants (p<0.05) but no significant difference was between H and M implants  **Mean total amount of**  **FGF-23** in P implants was significantly higher than in H implants  **25(OH)D3 total amount** was significantly lower in P implants than in H implants |
| Bhavsar  et al. ^58^  (2019)  USA | C-s | **Pa (H) 2**4  14F/10M  Age 64.54  **Pa (P)** 24  13F/11M  Age 68.21 | **H** 24  **P** 24 | >1 y | **H**: no inflammation, no attachment loss, no BL  **P**: inflammation, plaque or Sup, BoP,  PD≥4mm, BL>20%, but no>50% of the implant length | IL‐1β  MIP‐1α  MMP‐8 | Periopaper strips inserted for 15s into 4 sites (mesio‐buccal, disto-buccal,  mesio‐lingual/  palatal, disto‐lingual/  palatal) of each implant  Fluid amount evaluated with  Periotron.  Luminex IS‐100 and EMD Millipore | **IL‐1β (pg/ml)**  H: 1.7±2.9  P: 17.9±41.8  **MIP‐1α (pg/ml)**  H: 1.3±1.1  P: 1.2±1.2  **MMP‐8(ng/ml)**  H: 10.7±9.7  P: 12.1±1.2 | **IL‐1β** was significantly higher in P than in H implants (p=0.02)  **MIP‐1α** was similar between H and P implants  **MMP‐8** was slightly elevated in the P than in H implants |
| Chaparro  et al. ^59^  2020  Chile | C-s | **Pa(H)** 17  **Pa (M)** 19  **Pa (P)** 18  F/M NA  Age (30-78) | NA | NA | **H:** no swelling,  no BOP, no inflammation, no Sup, no increasing PD, no BL  **M:** peri-implant soft tissues inflammation, BOP, swelling, Sup, no BL  **P**: peri-implant soft tissues inflammation, BOP, swelling, Sup, progressive BL | MIP-3 α/ CCL-20  BAFF/  BLYS  RANKL  OPG | Periopaper strips inserted for 30 s into 4 sites (buccal, lingual/palatal, mesial, distal) of each implant.  Multiplex  Luminex assay | **Median (pg/mL)**  **MIP-3 α/ CCL-20**  H: 57.29(33.24-103.84)  M: 73.21 (27.68-115.41)  P: 48.845(29.16-83.82)  **RANKL**  H: 606.78  (584.06-612.46)  M: 595.42  (572.7-594.42)  P: 652.2  (629.5-765.62)  **OPG**  H: 1701(1361-2301)  M: 1701(1168-2194)  P: 2023.5(1462-3231)  **RANKL/OPG**  H: 0.29(0.26-0.43)  M: 0.37(0.24-0.58)  P: 0.31(0.21-0.56) | No statistically significant  differences in the levels of **MIP-3 α/ CCL-20** (p=0.802) and in  **RANKL/OPG ratio**  (p=0.770)  High levels of **RANKL** in P than in H implants  (p=0.003)  and in M than P (p=0.0002) implants |
| Algohar and Alqerban ^60^  (2020)  Saudi Arabia | C-s | **Pa (H)** 20  13F/7M  Age 34.4±4.2  **Pa (M)** 20  11F/9M  Age 37.6±4.8  **Pa (P)** 20  8F/12M  Age 42.5±6.4 | **H** 32  **M** 27  **P** 35 | **H** 38.6±8.4m  **M** 4.9±11.5m  **P** 51.8±14.2 | **H:** no sign of inflammation, no BoP, no increasing Pd, no BL  **M**: BoP and/or Sup with or without increased PD, no BL  **P**: BoP and/or Sup,  PD≥6 mm, bone level≥3 mm apical of the most coronal portion of intraosseous part of the implant after initial bone remodeling | PCT | Endodontic paper points.  ELISA | **(pg/ml)**  H: 7.33±4.18  M: 42.16±29.72  P: 119.86±74.54 | **PCT** levels were significantly higher in the M and P implants than in the H implants (p<0.001) and in the P than in the M implants  (p=0.001). |
| Hentenaar  et al. ^61^  (2021)  The Netherlands | C-s | **Pa (H)** 17  5F/12M  Age 63.9±17.6  **Pa(P)** 19  9F/10M  Age 56.5±11.5 | **H** 20  **P** 20 | ≥ 2 y | **H:** no BoP, no Sup, PPD<5mm, no BL  **P:** BoP and/or Sup, progressive BL≥2 mm compared to baseline Rx | IL-1β  IL-6  TNF-α  MCP-1  MIP-1α  MMP-8  OPG  G-CSF  sRANKL  IFN-γ | Periopaper strips inserted twice for  30 s into the sulcus/  pocket of each implant up to 1,2 µL  Fluid amount evaluated with  Periotron 8000.  Luminex™ Assay | **(Median, pg/mL)**  **IL-1β**  H: 390.5 (87.0;555.5)  P: 783.5 (414.0;2607.3)  **IL-6**  H: 20.3 (10.3;48.9)  P: 30.6 (10.6;67.4)  **TNF-α**  H: 11.3 (7.8;16.6)  P: 13.0 (10.5;20.4)  **MCP-1**  H: 48.13(26.9;72.9)  P: 58.2 (40.3;92.8)  **MIP-1α**  H: 15.63 (8.8;31.8)  P: 10.8(7.2;17.9)  **MMP-8**  H: 20,590.2 (13512.4;26929.4)  P: 34829.5 (24145.0;41791.5)  **OPG**  H: 34.3 (19.3;53.0)  P: 33.9 (20.3;66.2)  **G-CSF**  H: 0.0 (0.0;16.7])  P: 0.0 (0.0;24.0) | **IL-1β** (p=0.007) and **MMP-8** (p<0.001) median levels were significant higher  in P that in H implants  **TNF-α** (p=0.133), **IL-6** (p=0.402),  **G-CSF** (p=0.680),  **MIP-1α** (p=0.109),  **MCP-1** (p=0.136) and **OPG** (p=0.829) had comparable amounts in the PICF of H and P implants.  **sRANKL** and **INF-γ** were under the detection limit (levels under  7.40 pg/ml for sRANKL and under 14.40 pg/ml for **INF-γ**) |
| Jansson  et al. ^62^  (2021)  Sweden | C-s | **Pa** 163  91F/72M  Age 63±11.6  (29–83), | **H** 138  **P** 47 | ≥ 10 y  (10-15 y) | **H:** no BoP, no Sup, PPD<5mm, no BL  **P:** BoP/Sup, PD>4 mm, progressive BL≥2 mm | TNFSF13 (APRIL)  TNFSF13B (BAFF)  Chitinase-3-like-1  sIL6Rβ (gp130)  IFN-α2  IFN-β  sIL6Rα  IL-8  IL-11  IL-19  IL-20  IL-22  IFN-λ1 (IL-29)  IL-35  TNFSF14(LIGHT)  sTNF-R1  sTNF-R2  TNFSF12  (TWEAK) | Two periopaper strips inserted for 30s into the sulcus/  pocket of each implant.  Bio-Plex  Pro Human inflammation kit  Bio-Rad DC. Protein assay | **Median (25^th^;75^th^)**  **(pg/ml)**  **TNFSF13 (APRIL)**  H: 1.812(190;5,305)  P: 1,498(190;4,986)  **TNFSF13B (BAFF)**  H: 2,005 (1,270;3,173)  P: 4,903 (2,456;4,903)  **Chitinase-3-like-1**  H: 4,243 (1,855;8,346)  P: 11,762 (4,319;20,256)  **sIL6Rβ (gp130)**  H: 757 (486;1,223)  P: 1,575 (949;2,015)  **IFN-α2**  H: 2.3 (0.70;7.7)  P: 7.5 (3.4;14.3)  **IFN-β**  H: 18 (8.2;34)  P: 52 (16;88)  **sIL6Rα**  H: 276 (120;532)  P: 668 (337;1,327)  **IL−8**  H: 683 (340;1,373)  P: 2,894 (721;6,138)  **IL−11**  H: 0.97 (0.10;2.0)  P: 0.77 (0.05;2.1)  **IL−19**  H: IL−19  P: 38 (15;109)  **IL-20**  H: 35 (25;44)  P: 56 (32;73)  **IL−22**  H: 4.2 (1.1;10)  P: 13 (5.0;30)  **IFN-λ1 (IL-29)**  H: 1.6 (1.6;8.0)  P: 14 (2.7;26)  **IL-35**  H: 30 (12;50)  P: 34 (64;134)  **TNFSF14 (LIGHT)**  H: 60 (34;83)  P: 64 (26;89)  **sTNF-R1**  H: 299 (142;521)  P: 781 (400;1,420)  **sTNF-R2**  H: 75 (18;189)  P: 492 (92;1,349)  **TNFSF12 (TWEAK)**  H: 0.63 (0.50;10)  P: 21 (8.6;30) | **sIL6Rβ, sIL6Rα,**  and **IL-20 levels**  and the soluble receptors of **TNFα**, **sTNFR1**, and **sTNFR2**  were significantly higher in P than in H implant sites  (p≤0 .002) |
| Chaparro  et al. ^63^  (2021)  Chile | C-s | **Pa(H)** 17  12F/5M  Age 68.0  (48.0-78.0)  **Pa (M)** 19  12F/7M  Age 75.0  (48.0-77.0)  **Pa (P)** 18  11F/7M  Age 66.0  (54.0-69.0) | **H** 17  **M** 19  **P** 18 | **H**  60.0 m  (12.0-120.0)  **M** 48.0 m  (24.0-88.0)  **P** 48.0 m  (24.0-88.0) | **H:** no swelling,  no BOP, no inflammation, no Sup, no increasing PD, no BL  **M:** peri-implant soft tissues inflammation, BOP, swelling, Sup, no BL  **P**: peri-implant soft tissues inflammation, BOP, swelling, Sup, progressive BL | EVs  MVs  Exo  miRNA-21-3p  miRNA-150-5p  miRNA-26a-5p | Periopaper strips inserted for 30s into 4 sites (buccal, lingual/  palatal, mesial, distal) of each implant.  **EVs isolation**  Precipitation with ExoQuick system  **EVs**  **size** and **concentration**  nanoparticle tracking analysis  (NTA)  **EVs**  **size** and **morphology** transmission electron microscopy  (TEM)  **miRNA**  Qubit microRNA Assay Kit and  qPCR | **EVs (particles/ml)**  H: 0.3×10^9^  (0.2×10^9^-0.4×10^9^)  M: 0.6×10^9^  (0.2×10^9^-2.7×10^9^)  P: 0.8×10^9^  (0.4×10^9^-1.8×10^9^)  **MVs(particles/ml)**  H: 0.2x10^9^  (0.1×10^9^-0.4×10^9^)  M: 0.4×10^9^  (0.1×10^9^-0.1×10^9^)  P: 0.6×10^9^  (0.3×10^9^-1.0×10^9^)  **Exo (particles/ml)**  H: 0.1x10^9^  (0.1×10^9^-0.4×10^9^)  M: 0.4×10^9^  (0.1×10^9^-1.5×10^9^)  P: 0.5×10^9^  (0.2×10^9^-0.8×10^9^)  **miRNA-21-3p**  H: 0.1 (0.0-0.1)  M: 0.1 (0.0-0.1)  P: 0.0 (0.0-0.1)  **miRNA-150-5p**  H: 0.1 (0.1-0.2)  M: 0.2 (0.1-0.3)  P: 0.1 (0.1-0.3)  **miRNA-26a-5p**  H: 0.9 (0.4-1.0)  M: 0.6 (0.3-0.9)  P: 0.5 (0.3-0.7) | The total PICF concentration of **EVs** (p=0.023), **MVs** (p=0.002), and **Exo** (p=0.036) was significantly increased in P than in H implants. Expression of **miRNA-21-3p**  (p=0.011), and **miRNA-150-5p** (p=0.020) was significantly downregulated in P than in M implants |
| Alresayes  et al. ^64^  (2021)  Saudi Arabia | C-s | **Pa (H)** 44  22F/22M  Age 63.8±4.4  **Pa(P)** 44  20F/24M  Age 65.3±5.6 | **H** 63  **P** 68 | **H** 7.8±0.08 y  **P** 8.05±0.2 y | **P:** BoP and/or Sup, PD≥6mm,  BL≥3 mm | Cortisol | Periopaper strips inserted for 30s into the deepest buccal site of each implant  Fluid amount evaluated with  Periotron 8000.  ELISA | **Cortisol (pg/mL)**  H:79.2±8.4  P: 657.3±27.6 | **Cortisol** was significantly higher in P than in H implants  (p<0.001) |
| Gleiznys  et al. ^65^  (2021)  Lithuania | C-s | **Pa (H)** 30  **Pa (M)** 30  30F/30M  Age (55-70) | **H** 30  **M** 30 | 26.3±3.9 m | **H:** no MBI, no Sup, PD=0-3mm, no BL  **M**: MBI, no Sup,  PD=0-3mm, no BL | IL-17  TNF-α  MMP-8 | Capillary tube inserted for 30s into 2 or 3 sites of each implant.  **IL-17**  ELISA  **TNF-α**  Enzyme Amplified Sensitivity Immuno Assay (EASIA)  **MMP-8**  ELISA | **Median (25-75%)**  **IL-17**  H: 1.87 (0.83-3.249]  M: 17.94 (14.44-20.56)  **TNF-α**  H: 0.7 (0.53-0.74)  M: 3.08 (2.69-3.82)  **MMP-8**  H: 0.99 (0.68-1.4)  M: 3.27 (2.46-7.24) | **IL-17, TNF-a, and MMP-8** levels were significantly (p<0.001) higher in M than in H implants |
| Milinkovic  et al. ^66^  (2021)  Serbia | C-s | **Pa(H)** 35  17F/18M  Age 41.57±11.63  **Pa (M)** 45  30F/15M  Age 45.98±14.63  **Pa (P)** 50  28F/22M  Age 55.22±12.13 | **H** 35  **M** 45  **P** 50 | **H** 4.09±2.13y  **M**3.67±2.31y  **P** 7.58±3.41y | **H:** no clinical sign of inflammation,  no BOP and/or Sup, no increasing PD, no BL beyond bone level changes resulting from initial remodelling  **M:** BOP and/or Sup, increasing PD no BL beyond bone level changes resulting from initial remodelling  **P:** BOP and/or Sup, PD≥6mm, BL≥3mm apical of the most coronal portion of the intraosseous part of the implant | Notch 1  IL-6  IL-17 | Periopaper strips inserted for 30 s into the sulcus/  pocket of each implant.  ELISA | **Notch 1 (pg/ml)**  H: 1254.522±412.416  M: 1200.120±252.376  P: 1070.095±471.925  **IL-6 (pg/ml)**  H: 13.571±3.992  M: 27.352±52.606  P: 63.581±83.350  **IL-17 (pg/ml)**  H: 10.406±10.571  M: 52.824±155.533  P: 165.354±251.988 | **Notch 1** had a slight, not significant decrease in P than in M and H implants (p>0.05)  **IL-6** (p =0 .003) and **IL-17**  (p=0.002) had significantly higher levels in P than in H and M implants |
| Ali et al. ^67^  (2022)  Kuwait | C-s | **Pa (H)** 16  F/M NA  Age 52.2±1.2  **Pa (P)** 16  F/M NA  Age 52.7±1.6 | **H** 16  **P** 16 | NA | NA | Cortisol | Periopaper strips inserted for 30s into the mid-buccal site of each implant  Fluid amount evaluated with  Periotron 8000.  ELISA | **Cortisol (pg/ml)**  **H**: 73.08±8.53  **P**: 538.77±99.52 | **Cortisol** was significantly higher in P than in H implants  (p=0.001) |
| Lähteenmäk et al. ^68^  (2022)  Finland | C-c | **Pa (H)** 42  27F/15M  Age  71.45±7.67  (58-92)  **Pa (P)** 26  15F/11M  Age 68.77±9.89 (51-89) | **H** 42  **P** 26 | **H** 6.00±5.05y  (0-20)  **P** 7.46±3.06y  (3-12) | **H**: no BoP, PD<3mm, BL<2mm  **P**: BoP, PD≥3 mm, BL>2mm | MMP-8  Calprotectin  IL-6 | MMP-8 point-of-care (PoC) ipsticks inserted for 30s into the buccal site of each implant.  ELISA | **MMP-8 (ng/ml)**  H: 2.33±3.17  P: 4.62±3.16  **Calprotectin (ng/ml)**  H: 3999.62±3149.57  P: 7306.46±5241.21  **IL-6 (pg/ml**)  H: 0.66±1.15  P: 2.14±4.13 | Levels of all biomarkers were higher in P than in H implants |
| Chaparro  et al. ^69^  (2022)  Chile | C-s | **Pa (H)** 7  4F/3M  Age 73.8  (67-78)  **Pa (M)** 2  0F/2M  Age 61.5  (56-67)  **Pa (P)** 10  8F/2M  Age 67.8  (48-78) | **H** 7  **M** 2  **P** 10 | NA | Diagnosis of peri-implant health, peri-implant mucositis, and peri-implantitis was based on the 2017 EFP/AAP classification system for peri-implant health and diseases. | MIP-α/  CCL-20  BAFF/  BLyS  IL-23  RANKL  OPG | Periopaper strips inserted for 30s into the sulcus/  pocket of each implant.  Multiplex ELISA | **Median (interquartile range) (ng/mL)**  **MIP-3α/CCL-20**  H: 33.24(11.45–103.84)  M: 82.09(29.45–134.73)  P: 42.79 (13.25–149.26)  **BAFF/BLyS**  H: 9.7 (3.48–27.06)  M: 13.79 (10.36–17.23)  P: 17.065 (8.69–47.45)  **IL-23**  H: 70.16 (20.49–405.97)  M: 193.42(86.79–00.05)  P:134.905 (55.7–234.57)  **RANKL**  H: 629.5 (595.42–1168)  M: 830.35  (612.46–1048.24)  P: 640.84  (455.48–1003.12)  **OPG**  H: 2069  (1474.88–6071)  M: 2481.5  (1970–2993)  P: 1963  (885.88–4086) | **MIP-3α/CCL-20, BAFF/BLyS, IL-23**, and **RANKL** were higher in M and P than in H implants  **OPG** was higher in M than in H and P implants  **MIP-3α/CCL-20, IL-23, RANKL** and **OPG** were higher in M than in P implants  **BAFF/BLyS** was higher in P than in M implants |
| Swarup  et al. ^70^  (2022)  India | C-s | **Pa** 70  F/M NA  Age NA | **H**: 35  **P**: 35 | NA | **P**: GI≥2, BoP,  PD>6mm, BL | Calprotectin  NTx | Periopaper strips inserted for 30s into the sulcus/  pocket of each implant  Fluid amount evaluated with  Periotron.  ELISA | **Mean level (ng/site**)  **Calprotectin**  H: 43.3  P: 176.1  **NTx**  H: 2.92  P: 7.96  **Mean overall concentration ng/L)**  **Calprotectin**  H: 118.6  P: 241.9  **NTx**  H: 6.12  P: 9.98 | The overall mean **Calprotectin** and **NTx** values were significantly higher in P than in H sites (p= 0.05) |
| Ahmed  et al. ^71^  2022  Saudi Arabia | C-c | **Pa (H)** 15  F0/M15  Age  46.4 ± 7.2  **Pa (P)** 22  F0/M 15  Age  45 ± 10.2 | **H** 32  **P** 26 | **H** 45.6±3.4 m  **P** 48.1±9.8 m | NA | HMGB-1  TNF- α  IL-1ß | Periopaper strips inserted for 30s into the deepest PD of the buccal site of each implant  Fluid amount evaluated with  Periotron 8000.  ELISA | **Median/**  **interquartile range**  **HMGB-1 (pg/ml**)  H: 0.0 (0.0)  P: 143 (68)  **TNF- α (pg/ml**)  H: 33 (18)  P: 143 (68)  **IL-1ß (pg/ml)**  H: 28 (13)  P: 149 (76) | **HMGB-1, IL-1ß** and **TNF- α** levels were significantly higher in P than in H implants |
| Song  et al. ^72^  (2022)  China | C-s | **Pa** 14  6F/8M  Age 56.14  (33-67) | **H** 14  **P** 14 | > 1 y | **H**: no soft tissue inflammation no additional BL following initial healing  **P:** increasing PD and BL following initial healing, or PD≥6mm and BL≥3mm  without previous examination | G-CSF  IL-15  PDGF-AB/BB  IL-8  CXCL2  VEGF  IL-2  FGF-2  IL-1ß  IL-17A  RANTES  IL-6  IL-5  TGF-α  Eotaxin  TNF- α  IL-1α  IFN-ß  IL-1ra  MCP-1  GM-CSF  PD-L1/B7-H1  IL-12p70  IL-3  IL-17E  TRAIL  IL-7 | Periopaper strips inserted for 30s into 6 sites (mesio-buccal, mid-buccal, disto-buccal, mesio-lingual,  Mid-lingual and disto-lingual/  palatal) of each implant.  Luminex® Multiplex Assay | **Median (Q1;Q3)**  **(pg/mL)**  **G-CSF**  H: 73.9 (32.8;99.4)  P: 350.7(148.2;477.9)  **IL-15**  H: 1.3 (1.1;1.5)  P: 2 (1.6;2.2)  **PDGF-AB/BB**  H: 1.8 (1.6;2.1)  P: 3.4 (2.2;3.6)  **IL-8**  H: 2113 (864.3;2749.0)  P: 3533 (2363.5;3697.8)  **CXCL2**  H: 331.7 (214.1;672.8)  P: 1220.5 (726.3;1880.0)  **VEGF**  H: 324.7 (177.9;409.2)  P: 945.7 (456.8;1343.0)  **IL-2**  H: 14.9 (9.4;17.9)  P: 19.8 (17.3;21.0)  **FGF-2**  H: 15.5 (7.3;19.1)  P: 37.3 (23.4;61.7)  **IL-1ß**  H: 1403.0(735.8;2083.8)  P: 4311 (2388.5;5502.5)  **IL-17A**  H: 3.7 (3.0;4.4)  P: 10.5 (6.6;25)  **RANTES**  H: 108.7 (99.6;119.4)  P: 162.4 (122.2;191.7)  **IL-6**  H: 5.4 (4.5;8.2)  P: 38.3 (16.2;221.7)  **IL-5**  H: 1.9 (1.8;2.1)  P: 2.4 (2.1;2.5)  **TGF-α**  H: 52.7 (37.3;75.4)  P: 72.8 (65.7;88.1)  **Eotaxin**  H: 16.3 (13.7;18.1)  P: 19.5 (16.5;22.8)  **TNF-α**  H: 8.6 (4.7;9.1)  P: 11.3 (6.5;40.4)  **IL-1α**  H: 2593.5 (1814.7;4590.0  P: 6263.0 (4030.0;8863.0)  **IFN-ß**  H: 1.3 (1.1;1.9)  P: 2.4 (1.6;3.4)  **IL-1ra**  H: 18,813.0  (16,475.0;20,163.0)  P: 15,480.0  (14,761.0;17,251.0)  **MCP-1**  H: 50.0 (29.7;115.4)  P: 176.0 (72.5;415.1)  **GM-CSF**  H: 10.2 (8.0;18.9)  P: 25.7 (10.3;83.4)  **PD-L1/B7-H1**  H: 219.0 (133.0;248.8)  P: 141.1 (96.5;215.3)  **IL-12p70**  H: 6.8 (6.4;7.1)  P: 7.23 (6.6;8.3)  **IL-3**  H: 13.8 (6.2;27.4)  P: 5.7 (4.7;9.6)  **IL-17E**  H: 9.8 (9.5;10.6)  P: 11.8 (10.8;13.7)  **TRAIL**  H: 51.7 (34.7;71.4)  P: 71.1 (45.7;120.6)  **IL-7**  H: 1.1 (1.0;1.2)  P: 1.3 (1.3;1.0) | **IL-1ß** (p<0.001),  **IL-6** (p<0.001), and **IL-17A** (p=0.002) were significantly higher in P than in H implants  **IL-15, TNF-α**, and **IL-1α** showed comparable amounts between H and P implants (p<0.05)  **CXCL2** (p<0.001) and **G-CSF** (p<0.001)  were significantly higher in P than in H implants  **IL-8, RANTES, MCP-1, VEGF, PDGF-AB/BB**, and **FGF-2** were highly expressed in P than in H implants (p<0.05) |
| Ozgur  et al. ^73^  (2023)  Turkey | C-s | **Pa (H)** 23  F/M NA  Age  45.2 ± 12.8  **Pa (M)** 24  F/M NA  Age  54.08 ± 12.2  **Pa (P)** 22  F/M NA  Age  56.30 ± 7.08 | NA | **H** 6.08±2.27y  **M**6.50±2.28y  **P** 8.40±3.17y | **H:** no clinical inflammation (erythema, BoP, Sup), no BL  **M**: presence of clinical inflammation, no BL  **P:** PD>6mm, BL>3mm | IL-6  sST2 | Peripaper strips inserted for 30s into 2 sites (mesio-buccal, disto-buccal) of each implant  Fluid amount evaluated with  Periotron 8000.  ELISA | **Median (min-max)**  **IL-6 concentration (pg/ml)**  H: 102.27 (0.00-953.13)  M: 35.85 (0.00-391.75)  P: 58.52 (0.00-275.61)  **IL-6 total amount (pg/30s)**  H: 0.90 (0.00-6.14)  M: 2.49 (0.00-11.24)  P: 3.02 (0.00-14.98)  **sST2 concentration (pg/ml)**  H: 199.50 (0.00-627.27)  M: 166.10 (0.00-406.72)  P: 86.48 (0.00-314.06)  **sST2 total amount (pg/30s)**  H: 19.95 (0.00-35.14)  M: 26.81 (0.00-60.21)  P: 22.24 (0.00-40.43). | **sST2 total amount** was higher in M and P implants  **sST2 concentration** was higher in H than in M and P implants (p=0.043)  **IL-6 concentration** and **total amount** had no significant difference between implants |
| Ali et al. ^74^  (2023)  Kuwait | Cohort study | **Pa (H)** 22  8F/14M  Age  48.3 ± 1.05  **Pa (M)** 22  10F/12M  Age  54.6 ± 5.4  **Pa (P)** 22  8F/14M  Age  54.7 ± 3.7 | **H** 26  **M** 25  **P** 23 | **H** 5.5±1.7 y  **M** 4.5± 1.3y  **P** 8.9±2.04y | **H:** absence of gingival bleeding, erythema, swelling, and/or suppuration  **M**: erythema, gingival swelling, gingival bleeding, increased PD, BL>3mm.  **P:** erythema, gingival swelling, gingival bleeding, increased PD, BL≥3mm. | PGE_2_ | Peripaper strips inserted for 30s into mid-buccal site of each implant  Fluid amount evaluated with  Periotron 8000.  ELISA | **PGE_2_** **(µg/mL)**  H: 45.7±3.6  M: 103.1±15.4  P: 288.6± 15.3 | **PGE2 levels** were  significantly higher (p<0.01) in M than in H implants and in P than in M and H implants |

Abbreviations: C-s = cross-sectional; C-c = case-control; N/A= not available; PICF = peri-implant crevicular fluid; PISF = peri-implant sulcus fluid; Pa= patients; H = healthy; P = peri-implantitis; P-e= perimplantitis early; P-a= perimplantitis advanced; M = mucositis; M-e= mucositis early; M-a= mucositis advanced; PD = probing depth; GI = gingival index; sGI=simplified gingival index; PI = plaque index; mPI = modified plaque index; mBI=modified sulcus bleeding index; Bop= bleeding on probing; MBI= marginal bleeding index; Sup= suppuration; CAL=Clinical attachment loss; BL= bone loss; SBI= sulcus bleeding index
